# Pneumococcal Extracellular Serine Proteases: Molecular Analysis and Impact on Colonization and Disease

**DOI:** 10.3389/fcimb.2021.763152

**Published:** 2021-11-01

**Authors:** Murtadha Q. Ali, Thomas P. Kohler, Lukas Schulig, Gerhard Burchhardt, Sven Hammerschmidt

**Affiliations:** ^1^ Department of Molecular Genetics and Infection Biology, Interfaculty Institute of Genetics and Functional Genomics, Center for Functional Genomics of Microbes, University of Greifswald, Greifswald, Germany; ^2^ Department of Pharmaceutical and Medicinal Chemistry, Institute of Pharmacy, University of Greifswald, Greifswald, Germany

**Keywords:** *Streptococcus pneumoniae*, pneumococcal serine protease, respiratory infection, colonization, virulence factor, pathogenesis, structure

## Abstract

The pathobiont *Streptococcus pneumoniae* causes life-threatening diseases, including pneumonia, sepsis, meningitis, or non-invasive infections such as otitis media. Serine proteases are enzymes that have been emerged during evolution as one of the most abundant and functionally diverse group of proteins in eukaryotic and prokaryotic organisms. *S. pneumoniae* expresses up to four extracellular serine proteases belonging to the category of trypsin-like or subtilisin-like family proteins: HtrA, SFP, PrtA, and CbpG. These serine proteases have recently received increasing attention because of their immunogenicity and pivotal role in the interaction with host proteins. This review is summarizing and focusing on the molecular and functional analysis of pneumococcal serine proteases, thereby discussing their contribution to pathogenesis.

## Introduction

Pneumococci (*S. pneumoniae*, the pneumococcus) are Gram-positive, facultative anaerobic bacteria, colonizing asymptomatically the upper human respiratory tract (URT). Adherence to a mucosal surface of host tissues, predominantly indirectly *via* components of the extracellular matrix (ECM), is a prerequisite for establishing stable colonization (Bogaert et al., 2004). However, under certain circumstances, pneumococci disseminate from the nasopharynx to deeper tissues and the blood, leading to pneumonia and invasive diseases such as septicemia or meningitis (Song et al., 2013; Weiser et al., 2018; Bradshaw et al., 2020). Pneumococcal infections are a major cause of invasive diseases (invasive pneumococcal diseases, IPD) and death globally, especially in the most susceptible populations such as children, the elderly, and immunocompromised persons (O'Brien et al., 2009). The highest mortality is reported for children. Therefore, pneumococci are also called “The Forgotten Killer of Children,” as mentioned by UNICEF and WHO (UNICEF, 2006).

Pneumococci are endowed with a plethora of virulence factors contributing to adhesion, colonization, immune evasion, and host cell damage (Ljungh et al., 1996; Kadioglu et al., 2008; Voss et al., 2012; Weiser et al., 2018; Jahn et al., 2020). The initial steps of pneumococcal pathogenesis require an intimate, specific adherence to host structures and modulation of innate immune clearance mechanisms (Weiser et al., 2018). Pneumococcal adhesins recruit and bind to different human ECM and serum glycoproteins, including fibronectin, fibrinogen, vitronectin, thrombospondin-1, collagen, and plasmin(ogen) (Holmes et al., 2001; Bergmann et al., 2009; Voss et al., 2012; Fulde et al., 2013; Binsker et al., 2015). Striking examples are the multifunctional adhesins PspC (also referred to as CbpA), PavB, PsrP, and pilus type-1 (Rosenow et al., 1997; Pracht et al., 2005; Anderton et al., 2007; Kanwal et al., 2017). The close interaction of pneumococci with nasopharyngeal host cells is initially prevented by mucus and ciliary beating of the microvilli on the apical pole of mucosal epithelial cells (Clarke et al., 2011). However, pneumolysin inhibits ciliary beat frequency (Peter et al., 2017; Nishimoto et al., 2020), and enzymes like the pneumococcal neuraminidase NanA and hyaluronidase Hyl contribute to receptor exposure on the surface of host cells (Weiser et al., 2018). Importantly, pneumococci exhibit the ability to hijack host-derived serine protease proteolytic activities by binding plasmin(ogen), enabling ECM degradation, which facilitates colonization and dissemination of bacteria (Bergmann et al., 2005; Bergmann et al., 2013; Weiser et al., 2018). Proteases, especially serine proteases, are found in all living organisms. The intracellular and extracellular proteases are considered to be the most abundant and functional proteolytic enzymes (Page and Di Cera, 2008). These enzymes either hydrolyze peptide bonds within proteins or cleave them at their amino- or carboxyl-terminal ends (Patel, 2017). Bacterial proteases are involved in cell homeostasis, protein transport, and the structural integrity of the cell wall (Burchacka and Witkowska, 2016; Marquart, 2021). Many bacterial species express serine proteases that play a significant role in pathogenesis, such as *Bacteroides spp.*, *Clostridium spp., Pseudomonas aeruginosa*, and *Streptococcus* spp. (Macfarlane et al., 1988; Thibodeaux et al., 2007; de Stoppelaar et al., 2013; Martínez-García et al., 2018).

## Pneumococcal Proteases and Peptidases

*S. pneumoniae* expresses a wide range of proteases and peptidases, including cysteine proteases, zinc-metalloproteases, and serine proteases (Wani et al., 1996; Ishii et al., 2006; Marquart, 2021). More than 34 proteases in *S. pneumoniae* TIGR4 were recently reported and discussed (Kwon et al., 2011; Marquart, 2021). These proteases have different functions like involvement in the acquisition of nutrients, protein quality control, signal peptide cleavage for pre-protein secretion, and cleavage of host ECM proteins (Proctor and Manning, 1990; Marquart, 2021). It is reported that some proteases play a significant role in virulence (Collin and Olsén, 2003; Weiser et al., 2018; Kriaa et al., 2020). For instance, the zinc-metalloprotease ZmpA (also known as IgA1 protease) interacts with the host immune system by cleaving IgA into inactive components, and the zinc-metalloprotease ZmpB is important for the modification of pneumococcal surface proteins (Kilian et al., 1980; Novak et al., 2000).

## Pneumococcal Surface Proteins and Extracellular Serine Proteases

**Figure 1:**
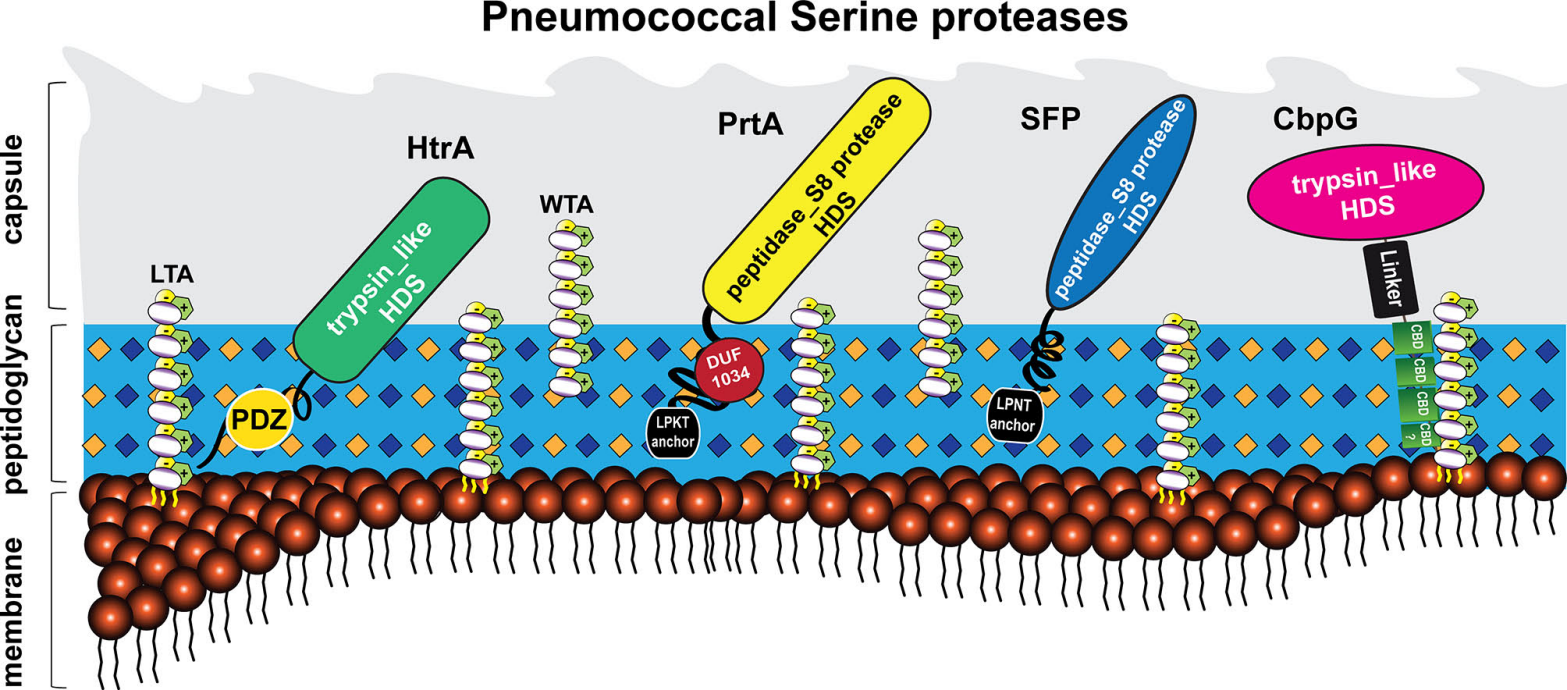
Localization of pneumococcal serine proteases on the bacterial surface. The pneumococcal cell wall of *S. pneumoniae* contains four different classes of surface-exposed proteins: choline-binding proteins (CBPs), sortase-anchored proteins containing a C-terminal LPXTG motif, lipoproteins, and non-classical surface proteins (Gamez and Hammerschmidt, 2012; Pribyl et al., 2014; Kohler et al., 2016). These proteins are associated with different structures of the cell wall, consisting of peptidoglycan (light blue), wall teichoic acids (WTA) and lipoteichoic acids (LTA) (carbohydrates repeating units in white circles for WTA and LTA). Wall teichoic acids are directly linked to the peptidoglycan (PGN), lipoteichoic acids are anchored to the phospholipid bilayer (membrane) *via* a lipid anchor (Tomasz, 1967). Pneumococcal teichoic acids are decorated with phosphorylcholine (*PCho*) residues (McCullers and Tuomanen, 2001). The pneumococcus displays four serine proteases on the bacterial surface (Mann et al., 2006; de Stoppelaar et al., 2013). Choline-binding protein G (CbpG, pink); bound non-covalently *via* the conserved choline-binding repeats (CBRs; green) to the phosphorylcholine residues of WTA or LTA. Subtilase family protein (SFP, blue) and cell wall-associated serine proteinase (PrtA, yellow) belong to the subtilisin-like proteases. Both proteins contain an N-terminal signal peptide and a C-terminal LPXTG motif. The latter is necessary to bind SFP and PrtA to the PGN, catalyzed by the transpeptidase Sortase A. High-temperature requirement A (HtrA, green) belongs to the family of trypsin-like proteases and contains no specific cell wall anchoring motif. All of these proteins contain the catalytic active domain with the Asp-His-Ser triad, which has proteolytic activity.

Besides in *S. pneumoniae*, serine proteases (or serine endopeptidases) have been found in many bacterial species such as *Haemophilus influenzae*, *Pseudomonas aeruginosa*, and other streptococcal species like *Streptococcus agalactiae* (group B streptococcus, GBS) (Male, 1979; Lyon and Caparon, 2004). Generally, the pneumococcus expresses different surface protein classes (Bergmann and Hammerschmidt, 2006; Pribyl et al., 2014; Kohler et al., 2016). Sortase-anchored proteins are covalently anchored to the peptidoglycan (PGN) *via* the sortase A, which cleaves a C-terminally located LPXTG motif (Bergmann and Hammerschmidt, 2006; Hammerschmidt, 2006; Nobbs et al., 2009; Löfling et al., 2011). In addition, the pneumococcal cell wall is decorated with up to 16 choline-binding proteins (CBPs), which are non-covalently bound to the phosphorylcholine of teichoic acids (Gosink et al., 2000). CBPs have been reviewed elsewhere (Maestro and Sanz, 2016). In this context, all pneumococcal serine proteases can be secreted and exposed on the pneumococcal cell surface, as shown in **Figure 1**. This extracellular localization enables a direct or indirect cleavage and inactivation of bound peptides, thereby leading to the degradation of specific substrates (Mann et al., 2006; Frolet et al., 2010). In fact, pneumococcal serine proteases are reported to play a crucial role in bacterial pathogenesis, such as adhesion, colonization, promotion of pneumococcal diseases, biofilm dispersal, and immune subversion of host cells (**Figure 4**) (Bergmann and Hammerschmidt, 2006; Moscoso et al., 2006; Mitchell and Mitchell, 2010; Voss et al., 2012; Pribyl et al., 2014; Chao et al., 2020; Ali et al., 2021).

The information on how pneumococcal serine proteases interfere with pathogenesis is crucial with respect to our understanding of pneumococci-host interactions. This review will focus on the four different pneumococcal serine proteases: HtrA, SFP, PrtA, and CbpG. These enzymes, encoded by genes of the core genome, are highly conserved and present among different pneumococcal serotypes (Bethe et al., 2001; Desa et al., 2008). The proteolytic activity is characterized by three amino acid (aa) residues, Ser-His-Asp, which form a so-called catalytic triad. The serine proteinase A (PrtA) and subtilase family protein (SFP) are cell wall-associated serine proteases of the S8 family of peptidases (Blum et al., 2021; Marquart, 2021). They are secreted and anchored covalently to the cell wall *via* the sortase A (Bethe et al., 2001; de Stoppelaar et al., 2013). PrtA contributes to host lung damage in a murine systemic infection model (de Stoppelaar et al., 2013; Mahdi et al., 2015), and in accordance, the gene encoding for PrtA is upregulated in the blood during acute pneumonia in mice (Bethe et al., 2001). In contrast, SFP may facilitate pneumococcal growth even after a lower infection dose in the lower respiratory tract (de Stoppelaar et al., 2013). The high-temperature requirement A (HtrA) serine protease is membrane-associated *via* an unknown mechanism and lacking a specific anchoring motif (Seol et al., 1991; Gasc et al., 1998; Fan et al., 2011), whereas CbpG is non-covalently associated with the wall teichoic (WTA) and lipoteichoic acids (LTA) (Mann et al., 2006). Previous studies suggested that CbpG could be a multifunctional protease playing an important role in mucosal colonization and sepsis (Mann et al., 2006). HtrA is a heat shock protein and chaperone involved in protein quality control, cell division, colonization, and virulence (Sebert et al., 2002; Ibrahim et al., 2004a; Cassone et al., 2012).

HtrA and PrtA are upregulated in the heat-dispersed population among the genetic variants (Pettigrew et al., 2014). We recently reported that the deficiency in three out of four serine proteases of TIGR4 with only one functional gene/protein or the deficiency of all serine proteases dramatically reduces adherence and nasopharyngeal colonization (Ali et al., 2021). Interestingly, the pneumococcal serine proteases are highly conserved among all pneumococcal serotypes and immunogenic (Bethe et al., 2001; Li et al., 2016; Hsu et al., 2018; Kazemian et al., 2018). Hence, serine proteases-driven pathogenesis is opening the avenue for new targets to develop specific antimicrobials. In this regard, our review presents a comprehensive summary of our current knowledge of pneumococcal serine proteases in order to gain insight into their potential roles in pneumococcal virulence and pathogenesis at a molecular level.

## Bioinformatics Analysis of Pneumococcal Serine Proteases

To characterize and compare pneumococcal serine proteases on the molecular level, different database tools including PSORT db 3.0 (Yu et al., 2011), multiple sequence alignment Clustal Omega (https://www.ebi.ac.uk/Tools/msa/clustalo/) and pairwise sequence alignment (https://www.ebi.ac.uk/Tools/psa/emboss_water/) were used. All analyzed serine protease gene sequences (*prtA* (*sp_0641*), *htrA* (*sp_2239*), and *cbpG* (*sp_0390*)) of *S. pneumoniae* strain TIGR4 or D39 for *SFP* (*spd_1753*) were retrieved from the KEGG database (Kanehisa et al., 2006). Signal sequences were predicted using the software tool SignalP 4.0 (Emanuelsson et al., 2007; Petersen et al., 2011). Choline-binding proteins are characterized by their typical choline-binding modules (CBM) consisting of characteristic choline-binding repeats (CBRs) (Maestro and Sanz, 2016). Moreover, for the prediction of transmembrane helices, the *TMHMM Server 2.0* algorithm (Hidden Markov Model for transmembrane protein topology prediction) was applied (Krogh et al., 2001). Functional domains were predicted using Pfam (Punta et al., 2012).

The genomes of 10 clinically relevant *S. pneumoniae* strains were analyzed on DNA and protein levels with BlastN and BlastP, respectively, for the homology analysis of pneumococcal serine proteases. The results revealed a maximum of four different serine proteases (NCBI, 2016). Comparisons on the protein level revealed high identities and similarities, indicating highly conserved sequences among the different pneumococcal strains (**Table 1**).

**Table 1:** Protein sequence homology [%] of serine proteases among different selected pneumococcal strains based on protein sequences from *S. pneumoniae* TIGR4 (Tettelin et al., 2001), and D39 for SFP.

*S. p.* Strain (serotype)		gene no.	CbpG	Gene no.	HtrA	Gene no.	PrtA	gene no.	SFP
**TIGR4 (4)**	**%ID**	*sp_0390*	**100.0**	*sp_2239*	**100.0**	*sp_0641*	**100.0**	*sp_1954*	**100.0**
	**%SIM**		**100.0**		**100.0**		**100.0**		**100.0**
**D39 (2)**	**%ID**	*spd_0356*	**99.0**	*spd_2068*	**100.0**	*spd_0558*	**95.8**	*spd_1753*	**100.0**
	**%SIM**		**99.5**		**100.0**		**97.8**		**100.0**
**EF3030 (19F)**	**%ID**	*EF3030_01920*	**99.6**	*EF3030_11105*	**99.7**	*EF3030_03025*	**97.5**	*—*	**—**
	**%SIM**		**99.6**		**100**		**98.7**		
**ST556 (19F)**	**%ID**	*snd:MYY_0470*	**87.0**	*snd:MYY_2162*	**100.0**	*snd:MYY_0688*	**95.9**	*—*	**—**
	**%SIM**		**95.7**		**100.0**		**97.9**		
**ST81 (23F)**	**%ID**	*spn23F03640*	**97.8**	*spn23F22720*	**99.7**	*spn23F05790*	**97.4**	*spn23F9760*	**100.0**
	**%SIM**		**98.5**		**100.0**		**98.8**		**100.0**
**JJA (14)**	**%ID**	*spj_0378*	**99.3**	*spj2269*	**99.7**	*spj_0592*	**97.4**	*spj_1948*	**100.0**
	**%SIM**		**100.0**		**100.0**		**98.6**		**100.0**
**R6 (2)**	**%ID**	*spr0349*	**99.0**	*spr2045*	**100.00**	*spr0561*	**95.8**	*spr1771*	**100.0**
	**%SIM**		**99.5**		**100.00**		**97.8**		**100.0**
**G54 (19F)**	**%ID**	*spg_0356*	**100.0**	*spg_2188*	**98.4**	*spg_0584*	**96.1**	*—*	**—**
	**%SIM**		**100.0**		**99.0**		**97.8**		
**Hungary 19A-6 (19A)**	**%ID**	*sph_0499*	**96.8**	*sph_2438*	**99.5**	*sph_0733*	**96.2**	*—*	**—**
	**%SIM**		**96.8**		**100.0**		**98.1**		
**R6_CIB17 (2)**	**%ID**	*—*	**—**	*E5Q10_10910*	**100.0**	*—–*	**—**	*E5Q10_09305*	**100.0**
	**%SIM**		**—**		**100.0**				**100.0**

Analysis of the proteins were performed with tool databases BlastP (NCBI, 2016), and EMBOSS (Rice et al., 2000). Protein sequences derived from TIGR4 strain were used as reference ID, Identity; SIM, Similarity. The meaning of the bold values are % ID, Identity percentage; % SIM, Similarity percentage.

## Molecular Characterization and Structure of Serine Protease-Like/Chaperone HtrA

HtrA belongs to the peptidase SA clan in the S1C family and is also identical to DO subfamily protease (Pallen and Wren, 1997). More than 180 members of these proteases, including HtrA, display trypsin-like protease characteristics (Pallen and Wren, 1997; NCBI, 2016). The family of these proteases combines a catalytic domain with at least one or more C-terminal PDZ domains (Lipinska et al., 1990), which is highly conserved in both pathogenic and nonpathogenic bacteria (Seol et al., 1991; Spiess et al., 1999; Backert et al., 2018). However, the first described HtrA protease in *E. coli* is known as DegP or DO protease and localized in the periplasmic space (Lipinska et al., 1990).

Bacterial HtrA is a heat-shock-induced serine protease that displays a multifunctional role like protein quality control and bacterial survival under different stress conditions such as oxidative and heat stress (Sebert et al., 2002; Singh et al., 2018). For instance, HtrA protease in *Lactococcus* is considered as a housekeeping protease (Poquet et al., 2000), while in other bacteria, HtrA prevents the cell from the cytotoxicity of misfolded proteins by refolding or degrading them (Clausen et al., 2002; Zarzecka et al., 2019). In *E. coli*, unlike other quality control proteins such as ClpXP, ClpAP, and HslUV, which need ATP for their chaperone function, HtrA is functional without ATP as an additional energy source (Clausen et al., 2011; Malet et al., 2012). More importantly, the function of HtrA proteins can be switched from chaperone to protease and the activity depends on the temperature (Spiess et al., 1999). The protease effect is in particular apparent at high temperatures ranging from 38-42°C, whereas the chaperon function is more pronounced at lower temperatures ranging from 30-37°C (Spiess et al., 1999).

In *S. pneumoniae*, HtrA is one of the best-studied and characterized serine proteases. High protein sequence identity (up to 100%) of the HtrA protein was detected in six different pneumococcal strains (**Table 1**) such as D39, Hungary 19A, serotype 19F_EF3030 and R6, indicating that HtrA is highly conserved. Therefore, it could be a desirable drug target to prevent pneumococcal diseases (Wessler et al., 2017; Xue et al., 2021). In pneumococci, HtrA is a surface-exposed serine protease, easily accessible for potential inhibitory substances or anti-infectives. HtrA is immunogenic and antibodies against HtrA are protective against invasive pneumococcal diseases (Li et al., 2016).

The molecular analysis of different HtrA serine proteases of other pathogenic bacteria *via* multiple sequence alignment (MSA) revealed a sequence similarity, especially in the functional protease and PDZ domains, as reviewed more extensively elsewhere (Backert et al., 2018; Boehm et al., 2018; Singh et al., 2018). They are widely distributed in many bacterial species such as *Escherichia coli*, *Legionella fallonii*, *Thermotoga maritima*, and *Mycobacterium tuberculosis* (Kim et al., 2003; Bai et al., 2011; Malet et al., 2012; Cortes et al., 2013; Chang, 2016; Singh et al., 2018).

Furthermore, we also analyzed the amino acid sequence of pneumococcal HtrA orthologs in other streptococci (**Table 2**). High sequence homologies of HtrA are present in *S. pyogenes* (group A streptococci), *S. agalactiae* (group B streptococci), *S. mitis*, and *S. mutans*. HtrA of *S. pyogenes* plays a significant role in cysteine protease streptococcal pyrogenic exotoxin B (SpeB) maturation and complement factor C5a cleavage (Lyon and Caparon, 2004; Cole et al., 2007). The deletion of HtrA in *S. mutans* enhanced the surface expression of several extracellular proteins such as glucan-binding protein GbpB and altered the biofilm formation (Biswas and Biswas, 2005).

**Table 2:** Comparison and distribution of pneumococcal serine proteases in other related bacterial species with amino acid sequence similarity and role in pathogenicity, updated from (Ali et al., 2021).

Protein (locus tag)	Proteinaccession no.	Bacterial species	Similarity [%]	Associated disease	Pathogenic function	Host-Targets	References
**PrtA, cell wall-associated serine protease**	AAK74791.1	*Streptococcus pneumoniae*	**100%**	CAP1, sepsis, meningitis	killing by apolactoferrin colonization adherence, pneumonia	cleaves human apolactoferrin, interact with collagen IV and plasminogen, cleavage of leader peptides from lantibiotics, possible adhesin	(Bethe et al., 2001; Mirza et al., 2011; Weiser et al., 2018; Ali et al., 2021)
PrtP (LP151), proteinase	M83946	*Lactobacillus paracasei*	**35.8%**	dental caries, rheumatic vascular disease, septicemia, and infective endocarditis	degrades secreted, cell-associated, and tissue-distributed and other proinflammatory chemokines	degrade proinflammatory chemokines	(von Schillde et al., 2012; Hörmannsperger et al., 2013)
PrtP (SK11), Proteinase, PIII-type	J04962, M26310	*Lactococcus lactis subsp. cremoris*	**35.4%**	Lactic acid bacteria (LAB), endocarditis chronic gastritis, central nervous infection	involved to adhesion and invasion, transit in the intestinal mucosa	adhesive properties, degrade alpha (S1)- and beta-caseins	(Nikolić et al., 2009; Inoue et al., 2014; Radziwill-Bienkowska et al., 2017)
PrtB, proteinase precursor	L48487	*Lactobacillus delbrueckii bulgaricus*,	**37.2%**	LAB Non-pathogenic	antibacterial activity, probiotic function,	cleaves beta-casein	(Abedi et al., 2013)
PrtH, cell envelope associated proteinase	AF133727	*Lactobacillus helveticus*	**36.5%**	LAB Nonpathogenic	antibacterial activity,	degrades alpha and beta-caseins	(Kunji et al., 1996)
PrtS, cell envelope proteinase	AAG09771	*Streptococcus thermophilus*	**35.3%**	LAB intestinal diseases	–	essential for growth	(Courtin et al., 2002)
ScpA, C5a peptidase	P15926	*Streptococcus pyogenes*	**38.1%**	necrotizing fasciitis, pharyngitis	facilitates the local infection	cleaves the human serum chemotaxis C5a	(Chmouryguina et al., 1996)
ScpB, C5a peptidase	U56908	*Streptococcus agalactiae*	**37.5%**	bacteremia, pneumonia	virulence factor, promote Fn-independent GAS invasion of human epithelial cells	inactivates C5a	(Bohnsack et al., 2000)
**CbpG, choline-binding protein G**	AAK74556.1	*Streptococcus pneumoniae*	**100%**	CAP, sepsis, meningitis	adherence, colonization virulence factor,	cell-attached form promotes adherence, extracellular form degrades fibronectin, important formucosal and invasive disease	(Gosink et al., 2000; Mann et al., 2006; Weiser et al., 2018; Ali et al., 2021)
GEJ60330 serine proteinase	GEJ60330.1	*Enterococcus faecalis*	**56%**	colonizing the gastrointestinal tract and oral cavity of animals and humans	endophthalmitis, peritonitis, endocarditis, and orthopaedic	–	(Thurlow et al., 2010)
serine protease	WP_010922847.1	*Staphylococcus aureus*	**40.4%**	CAP, bacteremia, endocarditis, osteomyelitis	involved in the evasion of host immunity	cleaves the ECM components	(Pietrocola et al., 2017)
serine protease	NP_460444.1	*Salmonella enterica subsp.*	**39.2%**	foodborne diseases (Salmonellosis)	epithelial cell invasion	cleavage of E‐cadherin	(Jajere, 2019)
Glu, endopeptidases	1P3C	*Bacillus intermedius*	**42.2%**	–	–	cleaves the peptide bond on the carboxyl end of glutamic acid	(Meijers et al., 2004)
**SFP, subtilisin-like serine protease**	ABC75782.1	*Streptococcus pneumoniae*	**100%**	CAP, sepsis, meningitis	facilitates bacterial growth, adherence, colonization	cleavage of leader peptides from lantibiotics	(de Stoppelaar et al., 2013; Ali et al., 2021; Marquart, 2021)
NisP, leader peptide-processing serine protease	4MZD_A	*Lactococcus Lactis*	**56.6%**	endocarditis infection	antibacterial lantibiotic	cleave leader peptides from lantibiotics	(Xu et al., 2014; Montalbán-López et al., 2018)
CspA, cell surface serine endopeptidase	CNG97209.1	*Streptococcus agalactiae*	**38.5%**	CAP, sepsis, meningitis	virulence factor, resistance to opsonophagocytosis	cleaves human fibronectin inactivates chemokines	(Harris et al., 2003; Bryan and Shelver, 2009)
**HtrA (DegP) serine protease/chaperone**	AAK76286.1	*Streptococcus pneumoniae*	**100%**	CAP, sepsis, meningitis	chaperone, heat-shock protein, protease, virulence factor, competence pathways, growth advantage in influenza A virus co-infection, adherence, colonization	quality control of secreted proteins	(Sebert et al., 2002; Ibrahim et al., 2004a; Ibrahim et al., 2004b; Cassone et al., 2012; de Stoppelaar et al., 2013; Kochan and Dawid, 2013; Sender et al., 2020; Ali et al., 2021)
	BAQ53883.1	*Streptococcus pyogenes*	**71.7%**	purulent diseases of the pharynx and skin	processing of extracellular virulence factors and hemolytic activity	cleavage of complement factor C5a	(Wexler et al., 1985; Lyon and Caparon, 2004)
	Q8DWP1	*S. agalactiae*	**73.6%**	bacteremia, pneumonia	–	–	
	VEI61035.1	*Streptococcus mutans*	**72.8%**	dental carries	colonization	biofilm formation	(Biswas and Biswas, 2005)
	WP_061099826.1	*Campylobacter jejuni*	**52.6%**	Campylobacteriosis, Guillain Barré syndrome	bacterial adhesion, transmigration, and invasion	cleavage of E-cadherin, apoptosis, and immune responses	(Zarzecka et al., 2020)
	AHC56659.1	*Helicobacter pylori*	**54.5%**	gastritis, ulcers symptoms	bacterial transmigration, activation of type IV secretion	cleavage of occludin, claudin‐8, E‐cadherin, and fibronectin	(Hoy et al., 2010; Schmidt et al., 2016; Tegtmeyer et al., 2017)
	5ZVJ_A	*Mycobacterium tuberculosis*	**52.7%**	tuberculosis	cell wall hydrolases	degrades a putative cell wall muramidase (Ami3)	(Wu et al., 2019)

Bold values means "Percentage identity".

**Figure 2:**
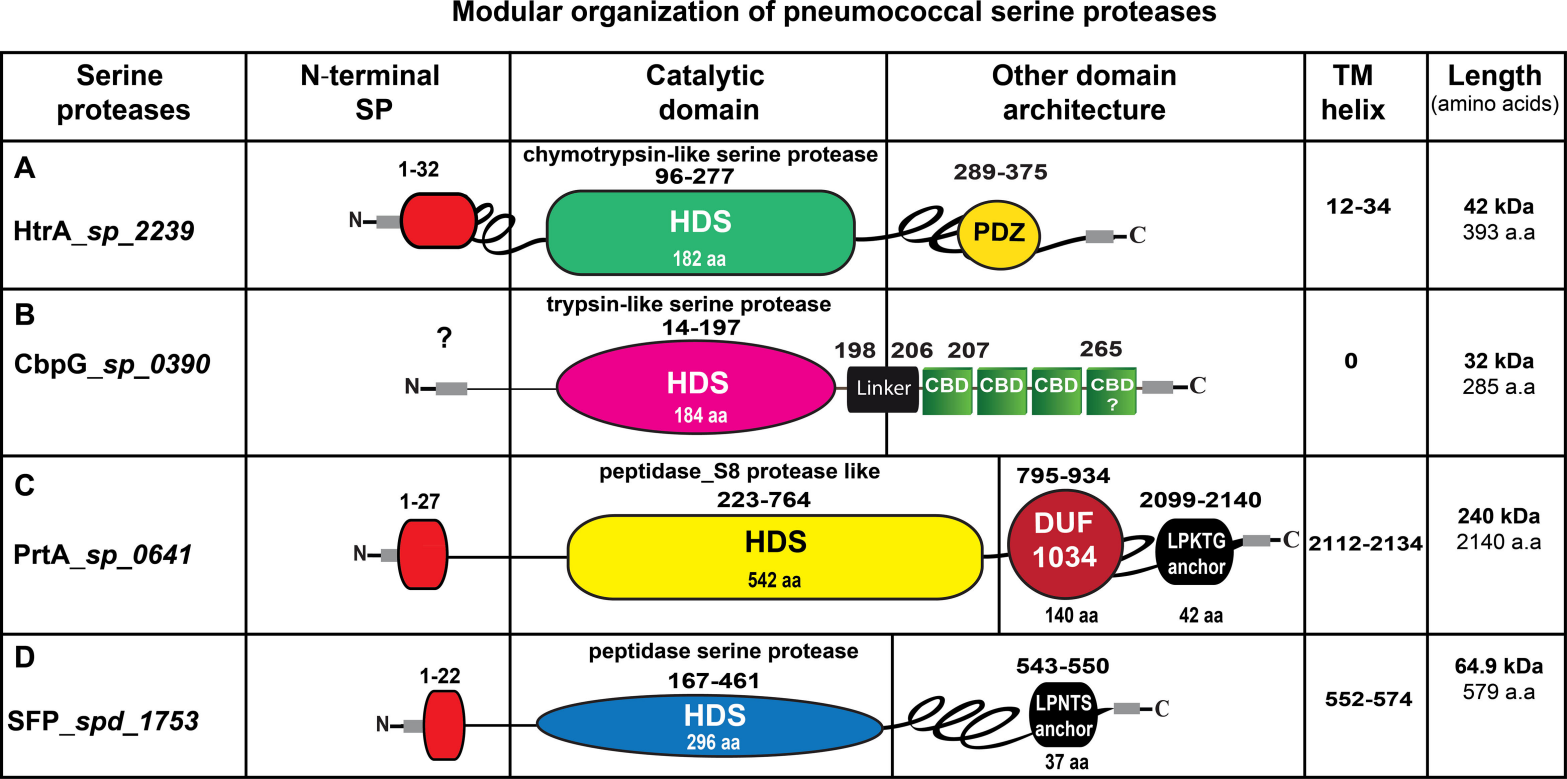
Schematic presentation of the modular organization in pneumococcal serine proteases. **(A)** HtrA (AAK76286.1), the signal peptide sequences (aa 1-32) are illustrated in red. The serine protease catalytic domain is shown in light green, PDZ domain is labeled in yellow. **(B)** CbpG (AAK74556.1), most likely has no signal peptide. The trypsin-like serine protease catalytic domain is shown in pink, the repeats of the choline-binding domains (CBDs) are marked in green, connected by short linker region aa 198-206. The C-terminal region aa 267-285 is probably also involved in binding to choline residues of teichoic acids. **(C)** PrtA (AAK74791.1), the signal peptide sequence aa 1-27 is depicted in red, the serine protease catalytic domain is illustrated in yellow, the DUF 1034 domain is shown in red. The C-terminal anchoring motif is labeled in yellow. **(D)** SFP (ABJ54257.1), the signal peptide sequence (1-22 aa) is shown in red, the serine protease catalytic domain is marked in blue. The length of each serine protease is given as the number of amino acids (aa). HDS, histidine, aspartate and serine; TM, transmembrane domain; SP, signal peptide sequences.

Besides the impact of HtrA on pneumococcal virulence, HtrA was shown to be a multifunctional protein involved in pneumococcal growth at higher temperatures, tolerance to oxidative stress, genetic transformation, regulation of bacteriocin production, and cell division (Dawid et al., 2009; Fan et al., 2010; Tsui et al., 2011). The pneumococcal HtrA protein (**Figure 2A**) contains an amino‐terminal signal peptide (31 aa), cleaved by signal peptidase I for secretion. The terminal signal peptide is followed by a single transmembrane helix domain (aa 12-34). Thus, HtrA is found on the surface and/or secreted from *S. pneumoniae* as predicted by the presence of a putative amino-terminal signal peptide (de Stoppelaar et al., 2013; Ali et al., 2021). Additionally, HtrA contains two highly conserved unique domains, a serine protease domain and a PSD-95/Dlg/ZO-1 (PDZ) domain (de Stoppelaar et al., 2013). The trypsin-like serine protease domain has the typical triad His^112^-Asp^152^-Ser^234^ (HDS) in the catalytic center, which was identified previously (de Stoppelaar et al., 2013) using Interproscan IPR009003 and IPR001940 (Zdobnov and Apweiler, 2001). Finally, the PDZ domain (abbreviation combining letters of the first three proteins discovered to share this domain, postsynaptic density protein, Drosophila discs large tumor suppressor, and zonula occludens-1) is located at the C-terminal end. HtrA in other bacterial species contains one or more PDZ domain(s) (Fan et al., 2011; Backert et al., 2018; Singh et al., 2018). In some situations, such as protein-protein interactions, the HtrA-PDZ domain acts as a protein folding stress sensor and controls the pyrolytic activity (Murwantoko et al., 2004; Wilken et al., 2004; Hasselblatt et al., 2007). Thus, the PDZ domains are responsible for recognizing and/or binding substrate proteins (Marquart, 2021). Fan et al. solved the pneumococcal HtrA-PDZ structure (Fan et al., 2011), which contains three α-helices and five β-strands (amino acid residues 262-386). Moreover, a comparison of the amino acid sequences of HtrA-PDZ domains in different bacterial species showed that the pneumococcal PDZ domain, which is most likely involved in the ligand recognition, has only a moderate sequence similarity and conserved secondary structure (Bohnsack et al., 2000).

The importance of HtrA in *S. pneumoniae* has been addressed in many studies. For instance, HtrA was shown to play an important role in pneumococcal competence, which is still challenging to understand due to the conflicting results. One study has shown that the HtrA protease is important for competence because the pneumococcal transformation efficiency was highly reduced in the *htrA-*mutant (Ibrahim et al., 2004b). In another study, the proteolytic activity analysis, which was performed with purified recombinant pneumococcal HtrA, revealed that HtrA cleaves the pneumococcal competence-stimulating peptide (CSP) *in vitro* (Cassone et al., 2012). Since CSP has a significant effect on pneumococcal transformation (Pestova et al., 1996), this fact suggests that HtrA has a considerable role in pneumococcal transformation efficiency and is needed for competence. In this study it has also been shown that the deletion of *htrA* or catalytic residues did not affect natural DNA competence (Cassone et al., 2012). However, the mutation strategies and transformation settings used in these two studies were different. It can be assumed that HtrA is necessary for the transformation process after the competence machinery is turned on by CSP. If the competence genes are expressed, CSP is not needed anymore and can be degraded by HtrA. Functional CSP seems to inhibit the transformation efficiency.

The specificity of HtrA toward CSP peptide degradation is based on a phenylalanine (nonpolar) residue. The addition of denatured bovine serum albumin (BSA) inhibits the CSP peptide from being cleaved by HtrA (Cassone et al., 2012). *S. pneumoniae* expresses several proteins contributing to competence, which are highly decreased during competence followed by stabilization with the exception of ComEA and ComEC. These membrane proteins are essential for pneumococcal transformation and responsible for DNA uptake (Liu et al., 2019). While *htrA*-mutants in the previous study have shown a lower transformation efficiency (Ibrahim et al., 2004b), ComEA or ComEC degradation was not evident. This suggests that HtrA plausibly degrades these proteins at later stages of competence (Liu et al., 2019). Last but not least, the regulation of HtrA seems to be dependent on bacterial culture conditions. It was shown that HtrA inhibits competence in a complex medium but not in a chemically defined medium (Petit et al., 2001). Overall, these findings show that HtrA acts as a competence regulator at the protein level and that environmental factors influence its regulation. Aside from the involvement of HtrA in competence, HtrA has been shown to be upregulated and controlled by the two-component regulatory system CiaRH (Sebert et al., 2002). A recent study showed that HtrA regulated by CiaRH is responsible for penicillin-binding protein 2x (PBP2x) degradation (Peters et al., 2021). In addition, HtrA is important for nasopharyngeal colonization and pneumococcal virulence (Sebert et al., 2002; de Stoppelaar et al., 2013; Ali et al., 2021).

## Molecular Analysis of the Serine Protease CbpG

The human pathogen *S. pneumoniae* expresses a special class of surface-proteins known as choline-binding proteins (CBPs). A common feature of this family of proteins is that they have a modular organization and are composed of at least two domains: a functional module (FM) and a choline-binding module (CBM). CBPs are found in pneumococci or closely related species (García et al., 1988; Sanz et al., 1992; Albrich et al., 2004; Blasi et al., 2012). The repetitive sequences of the CBM associate CBPs in a non-covalent manner to the cell wall by their interaction with phosphorylcholine residues of PGN-anchored WTA and membrane-anchored LTA (Pérez-Dorado et al., 2010; Maestro and Sanz, 2016). The CBM consists of three to eighteen repetitive sequences (CBRs) of about 20 amino acids (Pérez-Dorado et al., 2012; Galán-Bartual et al., 2015; Hilleringmann et al., 2015). Apart from LytB and LytC, the CBM is located in the C-terminal part of the protein, whereas the FM is located in the N-terminal region (Pérez-Dorado et al., 2010). The number of CBPs in *S. pneumoniae* ranges from 13 to 16 proteins and is strain-dependent (Gosink et al., 2000; Maestro and Sanz, 2016). Notably, CBPs play an essential role in the integrity of the cell wall, colonization processes, and interaction with host cells (Maestro and Sanz, 2016). Pneumococcal CbpG is a member of the CBP family, which also plays a significant role in pneumococcal mucosal colonization and during sepsis (Garcia-Bustos and Tomasz, 1987; Gosink et al., 2000). CbpG belongs to the peptidase S1, PA clan superfamily of peptidases, and is a trypsin-like serine protease (Kanz et al., 2005). The protein sequence indicates that this protein possesses a chymotrypsin-like fold and double β-barrel structure with a carboxyl-terminal choline-binding domain (NCBI, 2016; Yang et al., 2020). CbpG is considered to be a multifunctional surface-exposed serine protease with both proteolytic and adhesive functions (Gosink et al., 2000; Mann et al., 2006; Kazemian et al., 2018). These various functions of CbpG are necessary for the full virulence potential of *S. pneumoniae*. Such multifunctional proteinases can be found in many pathogenic bacterial species, and the C5a peptidase of group B streptococci (Beckmann et al., 2002; Cheng et al., 2002) and the well-characterized Pla surface protease from *Yersinia pestis* (Kukkonen and Korhonen, 2004) are striking examples.

**Figure 3:**
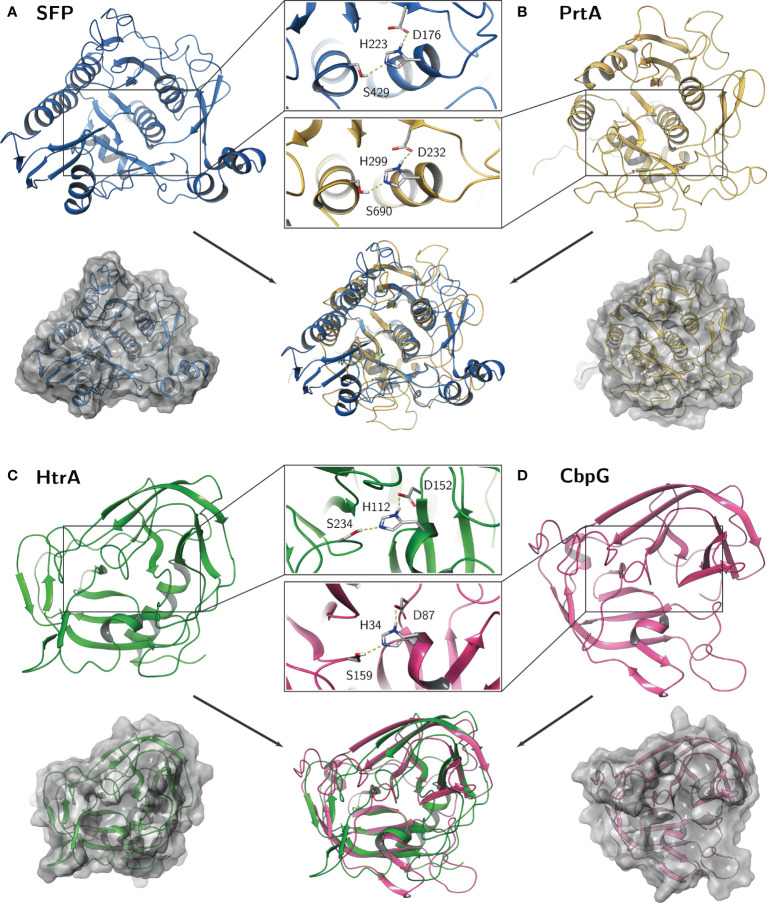
Predicted homology models of the pneumococcal serine proteases. Catalytic residues aspartate (D), histidine (H), and serine (S) are shown as sticks in detail for **(A)** SFP, **(B)** PrtA, **(C)** HtrA, and **(D)** CbpG. The calculations were performed within the Multiple Sequence Viewer/Editor application in Maestro (Schrödinger, 2020-4).

Depending on the pneumococcal strain and serotype, there are at least two variants of CbpG produced by pneumococci. The truncated variant without CBM is shortened due to a premature stop codon after the N-terminal catalytic functional module and found in D39 (serotype 2), Hungary19A-6 (19A), R6 (2) and ST556 (19F) (**Figure S1**). This variant is secreted and then released into the environment. In contrast, the full-length CbpG containing a CBM is cell wall-associated (Mann et al., 2006). The modular organization of CbpG (**Figure 2B**) shows that the catalytic residues are present independent of expressing a full-length protein, including a CBM or a truncated version without a functional CBM. In both configurations, the proteins exhibit proteolytic activity as confirmed earlier (Mann et al., 2006). Our genome re-analysis showed a high sequence identity and similarity of CbpG among various pneumococcal serotypes indicating CbpG is highly conserved and abundant among the different pneumococcal strains (**Table 1**). The molecular analysis of full-length CbpG (*sp_0390*) in TIGR4 (Tettelin et al., 2001) comprises 285 aa with a molecular weight of 32 kDa, as shown in **Figure 2B**. According to our SignalP 4.0 analysis, a leader peptide (secretion signal peptide) is not present in all analyzed serotypes except for serotype 19F strain ST556 (**Figure S1**). Therefore, it is still unknown whether and how CbpG is translocated from the cytoplasm to the bacterial cell surface. The functional domain is the trypsin-like domain with 184 aa spanning from aa 14-197, containing the catalytic triad His^34^-Asp^87^-Ser^159^ as predicted by the 3D structure analysis (**Figure 3**). Previous sequence analysis demonstrated 47% similarity of this domain to the S1 family of multifunctional surface-associated serine proteases (Mann et al., 2006). Furthermore, this domain is linked to the CBM by a short linker region (^aa^Lys-Pro-Phe-Ile^aa^) that provides flexibility to the protein and may provide stability to the catalytic domain. This catalytic functional module exhibits sequence similarities to trypsin-like serine proteases present in all CbpG variants (Gosink et al., 2000; Mann et al., 2006).

Moreover, it has been mentioned that the CBM, which is e.g., in strain TIGR4, exhibits only three choline-binding repeats (CBRs), which are located at position aa 207-265. This represents the shortest identified CBM among all choline-binding proteins. It has been proposed that at least four repeats are needed to attach the protein non-covalently to the teichoic acids of the cell wall (Yother and White, 1994) Therefore, it is still unknown if CbpG can bind to the bacterial cell surface when only three choline-binding repeats are present. In deletion studies of the CBM from the pneumococcal LytA amidase (Mellroth et al., 2014), it has been hypothesized that a higher number of CBRs leads to a higher affinity for teichoic acids of *S. pneumoniae* (Maestro and Sanz, 2016).

The CbpG amino acid sequence model was analyzed (Ali et al., 2021) and suggests that a fourth CBR at position aa 267-285 might attach CbpG to teichoic acids and allows the protein to be surface-associated. This repeat includes the aromatic residues YW and fulfills the number of aromatic residues involved in choline-binding (Waterhouse et al., 2009). The protein sequence homology of the CbpG to orthologues of other bacterial species was analyzed as well. Significant homologies of CbpG (40-56%) were found to serine proteinases of different bacterial species such as *Enterococcus faecalis*, *Staphylococcus aureus*, and *Salmonella enterica* (**Table 2**).

## Cell Wall-Associated Serine Protease PrtA

The protease PrtA belongs to the family of subtilisin-like proteases (also known as subtilases), which are part of S8 family peptidases (Bethe et al., 2001; Marquart, 2021). Pneumococcal PrtA is related to serine proteases present in lactococci, cleaving the amino-terminal leader sequences from lantibiotics (Blum et al., 2021). Lantibiotics are bacteriocin peptides that are bactericidal to outcompete other bacteria (Marquart, 2021). Interestingly, both, streptococci and lactococci exhibit a wide range of endopeptidase activity (Siezen, 1999).

In pneumococci, PrtA is a major surface serine protease involved in pneumococcal virulence (Zysk et al., 2000; Bethe et al., 2001). The role of PrtA in colonization and subsequent host invasion seems to be strain-specific (Mahdi et al., 2015). The first report on pneumococcal PrtA protease highlighted the immunogenicity because of its identification using convalescent-phase serum (Zysk et al., 2000). Interestingly, a previous study showed that PrtA is a highly conserved virulence factor in pneumococci and is found in almost all strains (Bethe et al., 2001). Both *in silico* analysis and flow cytometry confirmed that PrtA is surface localized (Wizemann et al., 2001).

The first molecular characterization of PrtA was done in 2001 (Bethe et al., 2001). Bethe and co-workers (Bethe et al., 2001) showed that pneumococci produce PrtA with different molecular weights. One variant produced by pneumococci has a molecular weight of 240 kDa, whereas a truncated form has only a molecular weight of 215 kDa, which cannot be explained by signal peptide cleavage only. The same observation was also found in the related proteases PrtP proteins of *Lactobacillus paracasei* and *Lactococcus lactis* (Vos et al., 1989; Holck and Naes, 1992). The full-length PrtA (strain TIGR4 *sp_0641*) form has a molecular weight of 240 kDa (2140 aa). The calculated mature form of PrtA has a molecular weight of 234 kDa after cleavage of the leader peptide and integration into peptidoglycan by sortase A. Furthermore, PrtA contains a typical sortase A recognition LPKTG motif spanning aa 2099-2140 followed by a hydrophobic region at the carboxy-terminus. The sortase A catalyzes covalent anchoring to the bacterial PGN (de Stoppelaar et al., 2013; Ali et al., 2021). PrtA consists of two domains, the active peptidase-S8 domain, which contains the typical catalytic triad (Asp^232^-His^299^- Ser^690^), spanning the region between aa 223-764 (Bethe et al., 2001; Ali et al., 2021). The second domain is a DUF-1034 (domain of unknown function), which consists of 140 amino acids and is localized between aa residues 795-934. The modular organization of PrtA is illustrated in (**Figure 2C**).

Of interest, the multisequence alignment of PrtA catalytic triad residues (Asp^232^-His^299^-Ser^690^) were highly homologous to other related bacterial species of subtilisin-like serine proteases. These catalytic triads showed a high degree of similarity and identity to the cell wall-associated proteases of *Streptococc*i, *Lactococc*i, and *lactobacilli* (Bethe et al., 2001; Bonifait et al., 2010).

Finally, the complete protein sequence homology to orthologues of other bacterial species was analyzed. As indicated in **Table 2**, PrtA shares significant similarities with other streptococcal subtilisin-like proteases. Interestingly, PrtA seems to be highly immunogenic in humans and mice; two segments of PrtA, the amino-terminal and carboxy-terminal thirds were found to be protective (Wizemann et al., 2001).

## Subtilase Family Protein SFP

The SFP serine protease (known as serine peptidase) is another enzyme able to cleave leader peptides from lantibiotics (Marquart, 2021). Similar to the PrtA protease, SFP belongs to subtilisin-like/or S8-family serine proteases. In *S. pneumoniae* D39 strain, SFP was identified as epidermin leader peptide processing serine protease EpiP (de Stoppelaar et al., 2013; Marquart, 2021).

The comparative analyses of *sfp* genes in *S. pneumoniae* strain D39 *spd_1753* (1740 nt, 579 aa), and TIGR4 *sp_1954* (1404 nt, 467 aa) was performed using the SYBIL database (Riley et al., 2011). The MSA analyses showed a shorter version of the *sfp* gene in TIGR4 compared to *sfp* of D39 and other strains (**Figure S2**). The truncation of *sfp* in strain TIGR4 is based on the deletion of one base (A) at position 1381. Instead of 8 A bases in a row, only seven are present in strain TIGR4, which was confirmed by DNA sequencing of the TIGR4 *sfp* gene. The generated frameshift leads to the premature stop at position 1404. Hence, this truncated SFP of TIGR4 cannot be covalently anchored to the peptidoglycan. Instead, TIGR4 SFP is secreted into the extracellular environment. However, these data have to be experimentally verified.

Based on the molecular characterization of SFP (de Stoppelaar et al., 2013; Ali et al., 2021), its secretion and protease activity has been predicted. The full-length SFP has a molecular weight of 64.9 kDa and exhibits an N-terminal signal peptide (aa 1-22) and a C-terminal LPNTG anchoring motif which is thought to be functional as a target site for the sortase A and anchoring the protein to PGN (Marraffini et al., 2006). In addition, the peptidase domain spanning the aa residues 167-461 contains the catalytic triad (Asp^176^-His^223^-Ser^429^) (**Figure 2D** and **Figure 3A**).

Furthermore, the genomic organization of the SFP locus in *S. pneumoniae* 19F and TIGR4/D39 strain is different. The *sfp* gene and six upstream and three downstream genes present in strain TIGR4 are not present in *S. pneumoniae* strain 19F EF3030 (Ali et al., 2021). Even more, the subtilisin-like protein SFP was not present in all the analyzed strains as observed by our *in silico* analysis (**Table 1**). Therefore, pneumococci have at least three serine proteases in the 19F_EF3030 strain (Ali et al., 2021), but probably four in most strains, such as D39 (serotype 2), TIGR4 (serotype 4), ST81 (serotype 23F), JJA (serotype 14), and R6 (serotype 2). However, the role of SFP in pneumococcal virulence is still unknown.

## Computer-Assisted 3D Structural Models of the Catalytic Domain of Serine Proteases

The HtrA of *E. coli* is well characterized and studied in detail for its functional role as chaperone and protease. The crystal structures of HtrA from *E. coli*, *Campylobacter jejuni* or *Termotoga maritima* showed that the active protease at elevated temperature is composed at least as a trimer by hydrophobic interaction of the subunits (Kim et al., 2003; Zarzecka et al., 2020). By using computer-assisted analysis, we compared the catalytic center of all four serine proteases from pneumococci. The calculations were performed within the Multiple Sequence Viewer/Editor application in Maestro (Schrödinger, 2020-4) using an energy-based approach. Templates were obtained by BLAST search in the PDB database (SFP: 4MZD; PrtA: 5FAX; HtrA: 5ZVJ; CbpG: 1P3C) (**Figures S3** **–** **6**). As mentioned, all serine proteases have in common the typical Ser-His-Asp triad, where the histidine is polarized through hydrogen bonding by aspartate, resulting in a polarization of serine and increased nucleophilicity of the hydroxyl oxygen atom. The highly conserved arrangement and distance between these three amino acids are crucial to form the catalytic center for the cleavage of peptide bonds.

On the one hand, the comparison revealed a quite similar catalytic domain structure between HtrA and CbpG with the common double Î²-barrel core motif adjacent to the catalytic triad. The Asp and Ser residues are localized on flexible loop structures, whereas the His residue is localized on a small helical fold (**Figure 3**). On the other hand, a similar subtilisin-like catalytic domain of SFP and PrtA was observed by this modeling. Here, the overall fold consists of a dominant 7-stranded parallel β-sheet, with the catalytic Asp on the first strand (S1) and five α-helices containing Ser and His. While the core catalytic motif seems quite similar, a protease-associated domain is found within the amino acid sequence of the PrtA catalytic domain, which may mediate protein-protein interactions or substrate specificity. Due to low sequence identity, it was omitted for the homology modeling and should be further explored. Because this is only a simplified view of the active proteolytic centers of these serine proteases, there are ongoing efforts to purify the recombinant serine proteases for X-ray crystallography.

## The impact of Pneumococcal Serine Proteases on Pneumococcal Pathogenesis

*S. pneumoniae* are versatile pathogens that modulate the immune response and circumvent host immune defense mechanisms. The enzymatic protease activity during pneumococcal infections can contribute to the destruction of the epithelial barrier or degradation of ECM components (Ljungh et al., 1996). Next, pneumococci try to establish a more severe infection by either transmigrating or/and disseminating to lungs, blood, middle ear, or the central nervous system.

Pneumococcal express various proteases and peptidases, which are involved in colonization, pneumonia, and septicemia (Marquart, 2021). In particular, pneumococcal serine proteases seem to play a role in invasive processes. In terms of specificity, substrates for serine protease are mainly the ECM component proteins, fibrin clots, cell membranes, and host immunomodulatory factors such as chemokines and cytokines (Frolet et al., 2010; Kim et al., 2010; Ruiz-Perez and Nataro, 2014).

Pneumococcal serine proteases might be involved in cleavage of adherence junctions or gap junction proteins to facilitate the pneumococcal paracellular route, which results in crossing of the epithelial barrier dissemination in the bloodstream. Recently, the impact of serine proteases on adherence, colonization, and subsequent virulence has been shown in various studies (**Table 2**).

The first description of various pneumococcal serine proteases along with their susceptibilities to different inhibitors was in 1991 (Courtney, 1991). Already at that time, their important role in pneumococcal pathogenesis had been reported and indicated by the degradation of host tissue components such as fibronectin, fibrinogen, elastin, laminin, and blood proteins. As has been mentioned before, pneumococcal serine proteases are virulence factors either secreted and/or bound to the bacterial cell surface. The benefit of expressing serine proteases is likely a higher efficiency in colonizing the nasopharyngeal cavity (Ali et al., 2021). To date, studies on pneumococcal serine proteases have been only marginally concentrated on their role in virulence-associated processes such as adhesion, colonization, or host defense evasion. Nevertheless, this section discusses the individual or combined impact of pneumococcal serine proteases HtrA, CbpG, PrtA, and SFP on pneumococcal colonization and how they contribute to host-pathogen interactions.

### The Extracellular HtrA Serine Protease Is Involved in Colonization and Invasive Disease

HtrA has been considered as one of the most important virulence factors associated with infectious diseases of various Gram-positive and Gram-negative bacteria. In general, HtrA protease significantly influences various functions such as bacterial fitness, adaptation to environmental stress, or enhance pneumococcal virulence (Murwantoko et al., 2004; Kochan and Dawid, 2013; Backert et al., 2018). Moreover, surface-exposed HtrA promotes nasopharyngeal colonization, whereas secreted HtrA facilitates the subsequent invasion of host tissue by degrading ECM components (Backert et al., 2018).

As mentioned above, HtrA is the best studied pneumococcal serine protease and was described for the first time 20 years ago. Subsequently, the influence of HtrA on pneumococcal pathogenesis has been addressed in several studies. For example, it has been shown that HtrA is upregulated and controlled by the two-component system (TCS) CiaRH (Sebert et al., 2002). Likewise, HtrA is considered one of the most critical serine proteases in pneumococcal virulence because HtrA degrades the competence stimulating peptides (CSPs), which impacts pneumococcal competence and late competence genes affect virulence (Ibrahim et al., 2004a; Ibrahim et al., 2004b; Cassone et al., 2012). Importantly, mice infection studies with *S. pneumoniae* D39 demonstrated that the deficiency of HtrA decreases bacterial load and inflammation in the lung after intranasal infection (de Stoppelaar et al., 2013).

Pneumococcal biofilms represent well-known pathophysiologically relevant conditions with a vital role in bacterial colonization, persistence and chronic infections (Domenech et al., 2012). In certain host compartments, pneumococci are protected against the attack of the immune system by forming sessile colonies embedded in an extracellular matrix of polysaccharides representing the biofilm. Recently, HtrA has been shown to modulate bacterial release (biofilm dispersal) from heat-induced biofilms, which were mimicking fever conditions (Chao et al., 2020).

**Figure 4:**
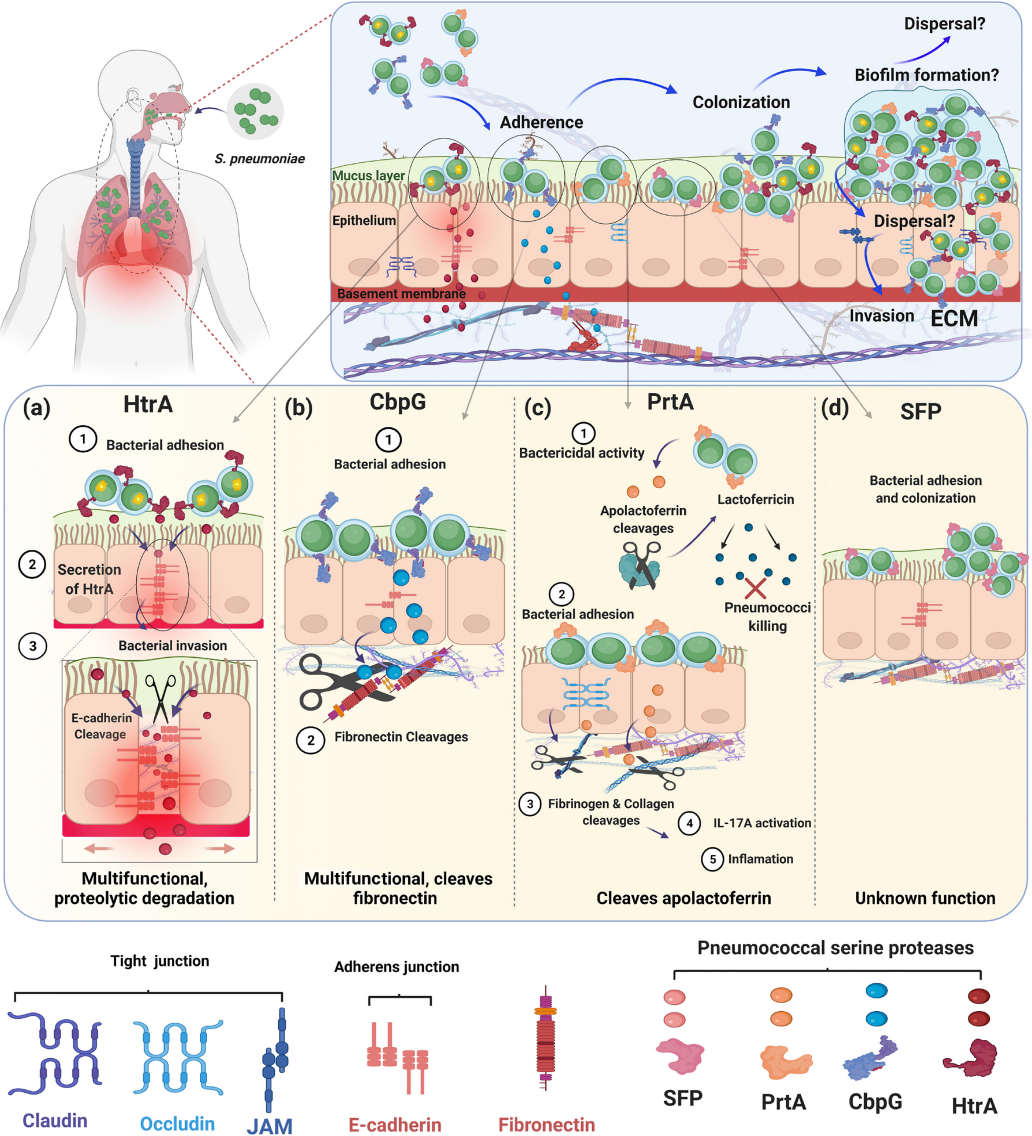
Role of pneumococcal serine proteases during adherence, colonization and invasion of the human host. The schematic models present different strategies of pneumococcal pathogenesis and the role of extracellular pneumococcal serine proteases. The upper panel shows that all four serine proteases HtrA, CbpG, PrtA, and SFP are involved in attachment to epithelial cells during nasopharyngeal colonization. Bacterial colonization and persistence in different host niches are dependent on the pneumococcal adherence capacity to host cells and tissues. Attachment to host cells facilitates bacterial cell aggregation and the formation of sessile communities like biofilms. Through the increased temperature during fever, released bacteria can switch from asymptomatic colonization to severe infections. The lower panels **(A)** HtrA, **(B)** CbpG, **(C)** PrtA, and **(D)** SFP show the individual role of each serine proteases for pneumococcal adherence and their potential to digest human ECM components. Figure created with Biorender.com.

During influenza-pneumococcal co-infections, HtrA induced the inflammation when highly expressed, thereby enhancing the bacterial load in a mouse pneumonia model (Sender et al., 2020). However, the underlying molecular mechanisms of how HtrA is implicated in colonization and invasion are not clearly understood. This raises the question of whether the HtrA protease degrades host proteins directly or do they have more complicated post-translational activities. The contribution of HtrA as chaperone or serine protease in pneumococcal attachment to epithelial cells and to deeper tissue is summarized in **Figure 4A**.

It is hypothesized that pneumococci can use the paracellular route to avoid intracellular killing and invade human host tissues (Iovino et al., 2016). To achieve this goal, pneumococci have to cleave proteins of adherences junction (AJ) and tight junctions (TJ) such as epithelial cadherin (E-cadherin), occludins, and claudins (Devaux et al., 2019). Interestingly, stimulation of Toll-like receptors (TLRs) during pneumococcal infections down-regulate claudins, facilitating pneumococci movement across the epithelium (Clarke et al., 2011). Furthermore, in human lungs that are infected with pneumococci, a reduction of alveolar occludin, ZO-1, claudin-5, and E-cadherin, was observed (Peter et al., 2017). Besides *S. pneumoniae*, many bacterial species possess a serine protease HtrA ortholog and the impact of HtrAs on bacterial pathogenesis was reviewed recently (Backert et al., 2018). Most of the HtrAs can cleave adherence junctions, tight junctions, and ECM proteins such as fibronectin and proteoglycans, leading to a disruption of the epithelial barrier and, this mode of action is, therefore, critical for the host cell damage (**Table 2**).

The serine protease HtrA of *Helicobacter pylori* represents a crucial secreted virulence factor (Schmidt et al., 2016). The disruption of the gastric epithelium leads to the transmigration of *H. pylori* across the epithelium and facilitates the oncogenic CagA protein injection into host cells. Consequently, HtrA can get into the extracellular space where it cleaves cell-to-cell junction factors, such as E-cadherin, leading to a disruption of the epithelial barrier, which then enables paracellular transmigration of the bacteria (Zawilak-Pawlik et al., 2019). E-cadherin belongs to the cell adhesion molecule superfamily (CAM) and represents the target of several pathogenic bacteria, which invade the host (Hulpiau and van Roy, 2009; Devaux et al., 2019). Interestingly, E-cadherin was described as an adherence receptor for the pneumococcal surface adhesin A (PsaA), which is also acting as a substrate-binding protein for manganese (Anderton et al., 2007).

Collectively, it seems that the involvement of HtrA in bacterial pathogenesis and the enzymatic activity of HtrAs have a common origin among (pathogenic) bacteria. Considering that bacterial HtrAs show high similarities, particularly their catalytic domain, two strategies are possible and may explain the functionality of HtrA. First, the surface localization of HtrA can significantly influence adherence and colonization as has been indicated earlier (Sebert et al., 2002; Ali et al., 2021). Second, HtrA undergoes the auto-cleavage process (Jomaa et al., 2009), and due to the secretion of HtrA into the environment, HtrA can degrade host components to facilitate invasion. These activities may explain data showing that a deficiency of HtrA in *S. pneumoniae* leads to a reduced bacterial load in the blood, liver, and spleen (Ibrahim et al., 2004b; de Stoppelaar et al., 2013). So far, it is not known if the pneumococcal HtrA can degrade occludins or E-cadherin. Therefore, further analysis is needed to prove that HtrA from pneumococci also cleaves E-cadherin and to determine other substrates of HtrA.

### The CbpG Serine Protease Cleaves ECM Proteins and Contributes to Adherence

Pneumococci must degrade the extracellular matrix to be able to disseminate in the host and cause invasive disease successfully. This requires the proteolytic activity of host acquired or self- proteases on the bacterial cell surface of the pneumococci. It is well known to date that several of the CBPs produced by pneumococci have multiple functions. The functions among CBPs are quite diverse, including proteolytic activity of the CbpG protein (Gosink et al., 2000). The importance of CbpG in pneumococcal pathogenesis is demonstrated by the fact that the gene encoding CbpG is upregulated in all *in vivo* niches (Mahdi et al., 2008). As mentioned above, *in silico* analysis of clinical isolates showed that *S. pneumoniae* express either a variant with a functional CBM attaching CbpG to the cell surface or a variant without a functional CBM leading to secretion of CbpG in the host environment (Mann et al., 2006). The truncated CbpG variant is nevertheless able to degrade ECM deposited fibronectin and casein *via* its trypsin-like serine protease similarly to the other variant (Mann et al., 2006). However, a functional CBM in the C-terminal part of CbpG is needed to contribute to pneumococcal adherence and colonization.

CbpG deficient pneumococci of strain 19F_EF3030 and TIGR4 showed a significant attenuation in *in vivo* rat or mice colonization models and reduced adherence to human epithelial cells (Mann et al., 2006; Ali et al., 2021). In addition, the mortality was reduced in a septicemia infection model with infant rats (Gosink et al., 2000). These studies indicated the importance of the serine protease CbpG as a factor modulating nasopharyngeal colonization and dissemination in the blood (Gosink et al., 2000; Ali et al., 2021). Therefore, CbpG could play a role in pneumococcal transition to the blood, which may be due to its fibronectin-cleaving potential (Mann et al., 2006).

The dual functions of CbpG, cleavage of host substrates and contributing to adherence to epithelial cells correlate with a substantial defect in the colonization of the nasopharynx by a *cbpG*-mutant (**Figure 4B**). On the one hand, one can also speculate that the proteolytic activity of CbpG on the bacterial cell surface can modify other pneumococcal surface proteins and enable them to interact with host cell receptors or soluble host proteins. On the other hand, CbpG probably modifies the ECM and eukaryotic cell surface, thereby facilitating adhesin-receptor interactions. These are still speculations and may also account for the other proteases. However, so far, no data are yet available supporting these ideas.

### Dual Role of Pneumococcal PrtA in Pneumococcal Pathogenesis

The cell wall-associated serine protease PrtA plays at least dual roles in pneumococcal infections. First, PrtA contributes to the cleavage of the human apolactoferrin to lactoferricin-like peptide, which serves as a cationic antimicrobial peptide and facilitates the killing of pneumococci (**Figure 4C**). This function is in a way surprising because it counteracts the virulence potential of pneumococci (Mirza et al., 2011). Second, PrtA is one of the largest pneumococcal surface proteins with a molecular weight of 240 kDa and is suggested to have adhesive functions similar to other sortase-anchored pneumococcal proteins (Frolet et al., 2010; Ali et al., 2021). A triple serine protease mutant of TIGR4 expressing only PrtA was significantly attenuated in the acute pneumonia model (Ali et al., 2021). This mutant is deficient in HtrA, and CbpG, which were shown be major virulence factors in pneumococcal pathogenesis (Mann et al., 2006; de Stoppelaar et al., 2013). In a systemic mouse infection model, mice infected with the *prtA*-mutant of strain D39 have extended survival times compared to wild-type infected mice (Bethe et al., 2001). The *prtA*-negative strain is significantly attenuated in an intranasal mouse infection model. Thus, expression of the gene encoding PrtA is confirmed to be upregulated in the blood (Mahdi et al., 2015). In addition, by applying the experimental nasopharyngeal mouse colonization model and using strain *S. pneumoniae* 19F it was shown that PrtA is necessary for an optimal colonization (Ali et al., 2021). More important, the use of a triple knockout in 19F lacking, therefore, all serine proteases, clearly indicated that serine proteases are indispensable for pneumococcal colonization (Ali et al., 2021).

Similar to other serine proteases PrtA degrades ECM components such as collagen IV and plasminogen, which suggests that this activity fosters pneumococcal transcytosis of the mucosal barrier and spread to the bloodstream (Frolet et al., 2010; Mahdi et al., 2015). PrtA was also shown to stimulate the IL-17A response, which is a significant mediator of tissue inflammation (Hsu et al., 2018). Although the impact of PrtA on pneumococcal colonization and invasive disease as well as its substrate specificities has to be explored in greater detail, the reported data are a strong hint for the importance of PrtA during colonization, inflammation, and invasive disease. Because PrtA is highly conserved and immunogenic, it might represent a promising candidate for a proteinaceous serotype-independent multi-component vaccine.

### The Unknown Functional Role of Pneumococcal Serine Protease SFP

The involvement of SFP in the pathogenesis of pneumococcal infections is still not apparent because of the minor effect of the *sfp*-mutant on virulence in experimental mouse infection models (de Stoppelaar et al., 2013).

SFP is not present in all pneumococcal strains and serotypes, as indicated in **Table 1**. However, the SFP protein shows high homology to the cell surface serine endopeptidase CspA (Bryan and Shelver, 2009), which is one of the important virulence factors for the human pathogen *Streptococcus agalactiae* (de Stoppelaar et al., 2013). Opsonophagocytosis of bacteria by host immune cells is one of the critical outcomes of classical complement activation (Harris et al., 2003). The complement component C3b deposited on the *S. agalactiae* cell surface can be cleaved by CspA, indicating the importance of CspA for immune evasion (Bryan and Shelver, 2009). So far, the impact of complement inactivation by its pneumococcal orthologue SFP is not known. In conclusion, the role of SFP for pneumococcal fitness, virulence, or immunomodulation needs further investigation and it will be interesting to identify SFP substrates.

## Conclusion and Future Perspectives

Serine proteases in pathogenic bacteria are, in general, key virulence determinants. In pneumococci, serine proteases have a function during colonization and pneumonia. This review article covers the molecular biology of pneumococcal serine proteases and their pivotal role in pathogenesis, starting from adherence, colonization, and immune evasion. Our *in silico* analysis in combination with hypothetical structural models revealed that the functional domains of pneumococcal serine proteases CbpG, HtrA, and PrtA, are highly conserved. The exception is SFP, which is produced only by a subset of strains. All serine proteases are secreted to the cell surface and depending on the variant, even released in the host environment. The 3D models show that the HtrA catalytic domain displays homologies to the CbpG catalytic domain, while SFP is quite similar to the catalytic domain of PrtA (**Figure 3**). Although all serine proteases have a typical catalytic triad, they might have different but also overlapping substrate specificities. The redundancy of serine proteases and probably their compensatory effect in the absence of one or more serine proteases makes it difficult to assess their individual contribution to pneumococcal fitness and virulence. Thus, all studies are in parts limited in their conclusions because of the redundancy of these serine proteases. This, in turn, leaves gaps of knowledge such as e.g., substrate specificities and host compartment specificities that have to be deciphered in experimental *in vivo* and advanced *in vitro* models. The immunogenicity of functional domains of pneumococcal serine proteases in combination with their highly conserved protein sequences fulfills one of the requirements for a protein-based serotype-independent (multi-) component vaccine. The individual potential as a vaccine candidate has, however, to be validated experimentally.

## Author Contributions

MA and SH conceived the concept for the review article. MA create the figures, drafted the work, and MA and GB performed the bioinformatic analyses. LS generated the 3D structural data and wrote this part. MA and SH wrote the review article, TK and GB revised it critically and gave final approval. All authors contributed to the article and approved the submitted version.

## Funding

This study was supported by the German Academic Exchange Service (DAAD) as a grant scholarship and part of the Ph.D. thesis of MA. Funding programme/-ID: Research Grants - Doctoral Programmes in Germany, 2017/18 (57299294), ST33. This study was also supported in part by the DFG (GRK 2719). The funders had no role in study design, decision to publish, or manuscript preparation.

## Conflict of Interest

The authors declare that the research was conducted in the absence of any commercial or financial relationships that could be construed as a potential conflict of interest.

## Publisher’s Note

All claims expressed in this article are solely those of the authors and do not necessarily represent those of their affiliated organizations, or those of the publisher, the editors and the reviewers. Any product that may be evaluated in this article, or claim that may be made by its manufacturer, is not guaranteed or endorsed by the publisher.
