# Supplementary material for: Pneumococcal Extracellular Serine Proteases: Molecular Analysis and Impact on Colonization and Disease
Source: Front Cell Infect Microbiol. 2021 Nov 1;11:763152. doi: 10.3389/fcimb.2021.763152 (PMC8592123; doi:10.3389/fcimb.2021.763152)
Supplement: Supplementary file 1 [file DataSheet_1.pdf]

Supplementary Material

Pneumococcal Extracellular Serine Proteases: Molecular Analysis and Impact on colonization and disease

Murtadha Q. Ali[1], Thomas P. Kohler[1], Lukas Schulig[2], Gerhard Burchhardt[1], and Sven Hammerschmidt[1*]

[1]Department of Molecular Genetics and Infection Biology, Interfaculty Institute of Genetics and Functional Genomics, Center for Functional Genomics of Microbes, University of Greifswald, Greifswald, Germany.

[2] Department of Pharmaceutical and Medicinal Chemistry, Institute of Pharmacy, University of Greifswald, Greifswald, Germany.

*** Correspondence:**
Prof. Dr. Sven Hammerschmidt
sven.hammerschmidt@uni-greifswald.de

```
Strain_gene no._(serotype)

ST556_snd:MYY_0470_(19F)       MKKTVYKKLGISIIASTLLASQLSTVSALSVISSTGEEYEVSETLQDSPGTNNFSLPNVS 60
Hungary19A-6_SPH_0499_(19A)    ------------------------------------------------------------ 0
D39_spd_0356_(2)               ------------------------------------------------------------ 0
R6_spr0349_(2)                 ------------------------------------------------------------ 0
ST81_spn23F03640_(23F)         ------------------------------------------------------------ 0
JJA_spj_0378_(14)              ------------------------------------------------------------ 0
TIGR4_sp_0390_(4)              ------------------------------------------------------------ 0
G54_spg_0356_(19F)             ------------------------------------------------------------ 0
EF3030_01920_(19F)             ------------------------------------------------------------ 0

ST556_snd:MYY_0470_(19F)       PTYGQYYTNQSEVIIGKNDLVKVKNTLQYPYSTSAYVESEFRGVKNGKDVTYRGSANFIK 120
Hungary19A-6_SPH_0499_(19A)    -----------------------------------------------MNVEGRGSVNFIK 13
D39_spd_0356_(2)               -----------------------------------MVLSKYYGVADGMNVEGRGSANFIK 25
R6_spr0349_(2)                 -------------------MIKIDNTLQYPYSTSAMVLSKYYGVADGMNVEGRGSANFIK 41
ST81_spn23F03640_(23F)         -----------------------------------------------MNVEGRGSANFIK 13
JJA_spj_0378_(14)              -----------------------------------------------MNVEGRGSANFIK 13
TIGR4_sp_0390_(4)              -----------------------------------MVLSKYYGVADGMNVEGRGSANFIK 25
G54_spg_0356_(19F)             -----------------------------------------------MNVEGRGSANFIK 13
EF3030_01920_(19F)             -----------------------------------------------MNVEGRGSANFIK 13
                                                                              :*   ***.****

ST556_snd:MYY_0470_(19F)       DNVLITAAHNIYKHEFGKDAEELYVTPARTPNSVPFGRIKVKEVRYLKEFRNLNSKDARE 180
Hungary19A-6_SPH_0499_(19A)    DNVLITAAHNYYRHDYGKEADDIYVLPAVSPSQELFGKIKVKEVCYLKEFRNLNSKDARE 73
D39_spd_0356_(2)               DNVLITAAHNYYRHDYGKEADDIYVLPAVSPSQELFGKIKVKEVRYLKEFRNLNSKDARE 85
R6_spr0349_(2)                 DNVLITAAHNYYRHDYGKEADDIYVLPAVSPSQELFGKIKVKEVRYLKEFRNLNSKDARE 101
ST81_spn23F03640_(23F)         DNVLITAAHNYYRHDYGKEADDIYVLPAVSPSQELFGKIKVKEVRYLKEFRNLNSKNARE 73
JJA_spj_0378_(14)              DNVLITAAHSYYRHDYGKEADDIYVLPAVSPSQEPFGKIKVKEVRYLKEFRNLNSKDARE 73
TIGR4_sp_0390_(4)              DNVLITAAHNYYRHDYGKEADDIYVLPAVSPSQEPFGKIKVKEVRYLKEFRNLNSKDARE 85
G54_spg_0356_(19F)             DNVLITAAHNYYRHDYGKEADDIYVLPAVSPSQEPFGKIKVKEVRYLKEFRNLNSKDARE 73
EF3030_01920_(19F)             DNVLITAAHNYYRHDYGKEADDIYVLPAVSPSQELFGKIKVKEVRYLKEFRNLNSKDARE 73
                               *********. *:*::**:*:::** ** :*..  **:****** ***********:***

ST556_snd:MYY_0470_(19F)       YDLALLILEEPIGAKLGTLGLPTSQKNLTGITVTITGYPSYNFKIHQMYTDKKQVLSDDG 240
Hungary19A-6_SPH_0499_(19A)    YDLALLILEEPIGAKLGTLGLPTSQKNLTGITVTITGYPSYNFKIHQMYTDKKQVLSDDG 133
D39_spd_0356_(2)               YDLALLILEKPIGAKLGTLGLPTSQKNLTGITVTITGYPSYNFKIHQMYTDKKQVLSDDG 145
R6_spr0349_(2)                 YDLALLILEKPIGAKLGTLGLPTSQKNLTGITVTITGYPSYNFKIHQMYTDKKQVLSDDG 161
ST81_spn23F03640_(23F)         YDLALLILEEPIGAKLGTLGLPTSQKNLTGITVTITGYLSYNFKIHQMYTDKKQVLSDDG 133
JJA_spj_0378_(14)              YDLALLILEEPIGAKLGTLGLPTSQKNLTGITVTITGYPSYNFKVHQMYTDKKQVLSDDG 133
TIGR4_sp_0390_(4)              YDLALLILEEPIGAKLGTLGLPTSQKNLTGITVTITGYPSYNFKIHQMYTDKKQVLSDDG 145
G54_spg_0356_(19F)             YDLALLILEEPIGAKLGTLGLPTSQKNLTGITVTITGYPSYNFKIHQMYTDKKQVLSDDG 133
EF3030_01920_(19F)             YDLALLILEEPIGAKLGTLGLPTSQKNLTGITVTITGYPSYNFKIHQMYTDKKQVLSDDG 133
                               *********:**************************** *****:***************

ST556_snd:MYY_0470_(19F)       MFLDYQVDTLEGASGSTVYDASNRVVGVHTLGDGANQINSAVKLNERNLPFIYSVLKGYS 300
Hungary19A-6_SPH_0499_(19A)    MFLDYQVDTLEGSSGSTVYDAS-RVVGVHTLGDGANQINSAVKLNERNLPFIYLFIRFLK 192
D39_spd_0356_(2)               MFLDYQVDTLEGSSGSTVYDASHRVVGVHTLGDGANQINSAVKLNERNLPFIYLFGS--- 202
R6_spr0349_(2)                 MFLDYQVDTLEGSSGSTVYDASHRVVGVHTLGDGANQINSAVKLNERNLPFIYLFGS--- 218
ST81_spn23F03640_(23F)         MFLDYQVDTLEGSSGSAVYDASHRVVGVHTLGDGANQINSAVKLNERNLPFIYSVLKGYS 193
JJA_spj_0378_(14)              MFLDYQVDTLEGSSGSTVYDASHRVVGVHTLGDGANQINSAVKLNERNLPFIYSVLKGYS 193
TIGR4_sp_0390_(4)              MFLDYQVDTLEGSSGSTVYDASHRVVGVHTLGDGANQINSAVKLNERNLPFIYSVLKGYS 205
G54_spg_0356_(19F)             MFLDYQVDTLEGSSGSTVYDASHRVVGVHTLGDGANQINSAVKLNERNLPFIYSVLKGYS 193
EF3030_01920_(19F)             MFLDYQVDTLEGSSGSTVYDASHRVVGVHTLGDGANQINSAVKLNERNLPFIYSVLKGYS 193
                               ************:***:***** ******************************   .

ST556_snd:MYY_0470_(19F)       LEGW-------------------------------------------------------- 304
Hungary19A-6_SPH_0499_(19A)    VTLLKDGRK--------------------------------------------------- 201
D39_spd_0356_(2)               ------------------------------------------------------------ 202
R6_spr0349_(2)                 ------------------------------------------------------------ 218
ST81_spn23F03640_(23F)         LEGWKKINGSWYYYRQHDKQTGWQEINDTWYYLDSSGKMLTDWQKVNGKWYYLNSNRAMV 253
JJA_spj_0378_(14)              LEGWKKINGSWYHYRQHDKQTGWQEINDTWYYLDSSGKMLTDWQKVNGKWYYLNSNGAMV 253
TIGR4_sp_0390_(4)              LEGWKKINGSWYHYRQHDKQTGWQEINDTWYYLDSSGKMLTDWQKVNGKWYYLNSNGAMV 265
G54_spg_0356_(19F)             LEGWKKINGSWYHYRQHDKQTGWQEINDTWYYLDSSGKMLTDWQKVNGKWYYLNSNGAMV 253
EF3030_01920_(19F)             LEGWKKINGSWYHYRQHDKQTGWQEINDTWYYLDSSGKMLTDWQKVNGKWYYLNSNGAMV 253
                                   CBD_1                CBD_2                CBD_3

ST556_snd:MYY_0470_(19F)       --------------------          304
Hungary19A-6_SPH_0499_(19A)    --------------------          201
D39_spd_0356_(2)               --------------------          202
R6_spr0349_(2)                 --------------------          218
ST81_spn23F03640_(23F)         TGSQTIDGKVYNFASSGEWI          273
JJA_spj_0378_(14)              TGSQTIDGKVYNFASSGEWI          273
TIGR4_sp_0390_(4)              TGSQTIDGKVYNFASSGEWI          285
G54_spg_0356_(19F)             TGSQTIDGKVYNFASSGEWI          273
EF3030_01920_(19F)             TGSQTIDGKVYNFASSGEWI          273
                                   CBD_?
```

Figure S1. Multiple sequence alignment comparisons of CbpG were performed using the bioinformatic tool "Clustal Omega" available on the following webpage: https://www.ebi.ac.uk/Tools/msa/clustalo/. The signal peptide was detected by using the software tool SignalP 4.0 (http://www.cbs.dtu.dk/services/SignalP-4.0/), and marked in red color. The repeats of the CBM at the C-terminal region are highlighted by different colors.

Strain_*gene no.*_(serotype)

```
TIGR4_sp_1954_(4)              ---MKKKYWTLAILFFCLFNNSVTAQEIPKNLDGNITHTQTSESFSESDEKQVDYSNKNQ     57
D39_spd_1753_(2)               MKIMKKKYWTLAILFFCLFNNSVTAQEIPKNLDGNITHTQTSESFSESDEKQVDYSNKNQ     60
ST81_spn23F19760_(23F)         MKIMKKKYWTLAILFFCLFNNSVTAQEIPKNLDGNITHTQTSESFSESDEKQVDYSNKNQ     60
JJA_spj_1948_(14)              MKIMKKKYWTLAILFFCLFNNSVTAQEIPKNLDGNITHTQTSESFSESDEKQVDYSNKNQ     60
R6_spr1771_(2)                 MKIMKKKYWTLAILFFCLFNNSVTAQEIPKNLDGNITHTQTSESFSESDEKQVDYSNKNQ     60
R6_E5Q10_09305_CIB17_(2)       MKIMKKKYWTLAILFFCLFNNSVTAQEIPKNLDGNITHTQTSESFSESDEKQVDYSNKNQ     60
                                  *********************************************************

TIGR4_sp_1954_(4)              EEVDQNKFRIQIDKTELFVTTDKHLEKNCCKLELEPQINNDIVNSESNNLLGEDNLDNKI     117
D39_spd_1753_(2)               EEVDQNKFRIQIDKTELFVTTDKHLEKNCCKLELEPQINNDIVNSESNNLLGEDNLDNKI     120
ST81_spn23F19760_(23F)         EEVDQNKFRIQIDKTELFVTTDKHLEKNCCKLELEPQINNDIVNSESNNLLGEDNLDNKI     120
JJA_spj_1948_(14)              EEVDQNKFRIQIDKTELFVTTDKHLEKNCCKLELEPQINNDIVNSESNNLLGEDNLDNKI     120
R6_spr1771_(2)                 EEVDQNKFRIQIDKTELFVTTDKHLEKNCCKLELEPQINNDIVNSESNNLLGEDNLDNKI     120
R6_E5Q10_09305_CIB17_(2)       EEVDQNKFRIQIDKTELFVTTDKHLEKNCCKLELEPQINNDIVNSESNNLLGEDNLDNKI     120
                               ************************************************************

TIGR4_sp_1954_(4)              KENVSHLDNRGGNIEHDKDNLESSIVRKYEWDIDKVTGGGESYKLYSKSNSKVSIAILDS     177
D39_spd_1753_(2)               KENVSHLDNRGGNIEHDKDNLESSIVRKYEWDIDKVTGGGESYKLYSKSNSKVSIAILDS     180
ST81_spn23F19760_(23F)         KENVSHLDNRGGNIEHDKDNLESSIVRKYEWDIDKVTGGGESYKLYSKSNSKVSIAILDS     180
JJA_spj_1948_(14)              KENVSHLDNRGGNIEHDKDNLESSIVRKYEWDIDKVTGGGESYKLYSKSNSKVSIAILDS     180
R6_spr1771_(2)                 KENVSHLDNRGGNIEHDKDNLESSIVRKYEWDIDKVTGGGESYKLYSKSNSKVSIAILDS     180
R6_E5Q10_09305_CIB17_(2)       KENVSHLDNRGGNIEHDKDNLESSIVRKYEWDIDKVTGGGESYKLYSKSNSKVSIAILDS     180
                               ************************************************************

TIGR4_sp_1954_(4)              GVDLQNTGLLKNLSNHSKNYVPNKGYLGKEEGEEGIISDIQDRLGHGTAVVAQIVGDDNI     237
D39_spd_1753_(2)               GVDLQNTGLLKNLSNHSKNYVPNKGYLGKEEGEEGIISDIQDRLGHGTAVVAQIVGDDNI     240
ST81_spn23F19760_(23F)         GVDLQNTGLLKNLSNHSKNYVPNKGYLGKEEGEEGIISDIQDRLGHGTAVVAQIVGDDNI     240
JJA_spj_1948_(14)              GVDLQNTGLLKNLSNHSKNYVPNKGYLGKEEGEEGIISDIQDRLGHGTAVVAQIVGDDNI     240
R6_spr1771_(2)                 GVDLQNTGLLKNLSNHSKNYVPNKGYLGKEEGEEGIISDIQDRLGHGTAVVAQIVGDDNI     240
R6_E5Q10_09305_CIB17_(2)       GVDLQNTGLLKNLSNHSKNYVPNKGYLGKEEGEEGIISDIQDRLGHGTAVVAQIVGDDNI     240
                               ************************************************************

TIGR4_sp_1954_(4)              NGVNPHVNINVYRIFGKSSASPDWIVKAIFDAVDDGNDIINLSTGQYLMIDGEYEDGTND     297
D39_spd_1753_(2)               NGVNPHVNINVYRIFGKSSASPDWIVKAIFDAVDDGNDIINLSTGQYLMIDGEYEDGTND     300
ST81_spn23F19760_(23F)         NGVNPHVNINVYRIFGKSSASPDWIVKAIFDAVDDGNDIINLSTGQYLMIDGEYEDGTND     300
JJA_spj_1948_(14)              NGVNPHVNINVYRIFGKSSASPDWIVKAIFDAVDDGNDIINLSTGQYLMIDGEYEDGTND     300
R6_spr1771_(2)                 NGVNPHVNINVYRIFGKSSASPDWIVKAIFDAVDDGNDIINLSTGQYLMIDGEYEDGTND     300
R6_E5Q10_09305_CIB17_(2)       NGVNPHVNINVYRIFGKSSASPDWIVKAIFDAVDDGNDIINLSTGQYLMIDGEYEDGTND     300
                               ************************************************************

TIGR4_sp_1954_(4)              FETFLKYKKAIDYANQKGVIIVAALGNDSLNVSNQSDLLKLISSRKKVRKPGLVVDVPSY     357
D39_spd_1753_(2)               FETFLKYKKAIDYANQKGVIIVAALGNDSLNVSNQSDLLKLISSRKKVRKPGLVVDVPSY     360
ST81_spn23F19760_(23F)         FETFLKYKKAIDYANQKGVIIVAALGNDSLNVSNQSDLLKLISSRKKVRKPGLVVDVPSY     360
JJA_spj_1948_(14)              FETFLKYKKAIDYANQKGVIIVAALGNDSLNVSNQSDLLKLISSRKKVRKPGLVVDVPSY     360
R6_spr1771_(2)                 FETFLKYKKAIDYANQKGVIIVAALGNDSLNVSNQSDLLKLISSRKKVRKPGLVVDVPSY     360
R6_E5Q10_09305_CIB17_(2)       FETFLKYKKAIDYANQKGVIIVAALGNDSLNVSNQSDLLKLISSRKKVRKPGLVVDVPSY     360
                               ************************************************************

TIGR4_sp_1954_(4)              FSSTISVGGIDRLGNLSDFSNKGDSDAIYAPAGSTLSLSELGLNNFINAEKYKEDWIFSA     417
D39_spd_1753_(2)               FSSTISVGGIDRLGNLSDFSNKGDSDAIYAPAGSTLSLSELGLNNFINAEKYKEDWIFSA     420
ST81_spn23F19760_(23F)         FSSTISVGGIDRLGNLSDFSNKGDSDAIYAPAGSTLSLSELGLNNFINAEKYKEDWIFSA     420
JJA_spj_1948_(14)              FSSTISVGGIDRLGNLSDFSNKGDSDAIYAPAGSTLSLSELGLNNFINAEKYKEDWIFSA     420
R6_spr1771_(2)                 FSSTISVGGIDRLGNLSDFSNKGDSDAIYAPAGSTLSLSELGLNNFINAEKYKEDWIFSA     420
R6_E5Q10_09305_CIB17_(2)       FSSTISVGGIDRLGNLSDFSNKGDSDAIYAPAGSTLSLSELGLNNFINAEKYKEDWIFSA     420
                               ************************************************************

TIGR4_sp_1954_(4)              TLGGYTYLYGNSFAAPKVSGAIAMIIDKYKLKDQPYNYMFVKKFWKKHYQ----------     467
D39_spd_1753_(2)               TLGGYTYLYGNSFAAPKVSGAIAMIIDKYKLKDQPYNYMFVKKILEETLPVKNGIKVLNI     480
ST81_spn23F19760_(23F)         TLGGYTYLYGNSFAAPKVSGAIAMIIDKYKLKDQPYNYMFVKKILEETLPVKNGIKVLNI     480
JJA_spj_1948_(14)              TLGGYTYLYGNSFAAPKVSGAIAMIIDKYKLKDQPYNYMFVKKILEETLPVKNGIKVLNI     480
R6_spr1771_(2)                 TLGGYTYLYGNSFAAPKVSGAIAMIIDKYKLKDQPYNYMFVKKILEETLPVKNGIKVLNI     480
R6_E5Q10_09305_CIB17_(2)       TLGGYTYLYGNSFAAPKVSGAIAMIIDKYKLKDQPYNYMFVKKILEETLPVKNGIKVLNI     480
                               *******************************************: ::

TIGR4_sp_1954_(4)              ------------------------------------------------------------     467
D39_spd_1753_(2)               PNVLRYDLNMLQLEYKNEQSWDSFIDNVNLIELEERIQTTIGIKQINTHNIITIAREGYS     540
ST81_spn23F19760_(23F)         PNVLRYDLNMLQLEYKNEQSWDSFIDNVNLIELEERIQTTIGIKQINTHNIITIAREGYS     540
JJA_spj_1948_(14)              PNVLRYDLNMLQLEYKNEQSWDSFIDNVNLIELEERIQTTIGIKQINTHNIITIAREGYS     540
R6_spr1771_(2)                 PNVLRYDLNMLQLEYKNEQSWDSFIDNVNLIELEERIQTTIGIKQINTHNIITIAREGYS     540
R6_E5Q10_09305_CIB17_(2)       PNVLRYDLNMLQLEYKNEQSWDSFIDNVNLIELEERIQTTIGIKQINTHNIITIAREGYS     540

TIGR4_sp_1954_(4)              ---------------------------------------     467
D39_spd_1753_(2)               QNYLPNTSENTYNSLQVSLVGVLLLFISMVNILWAKKSK     579
ST81_spn23F19760_(23F)         QNYLPNTSENTYNSLQVSLVGVLLLFISMVNILWAKKSK     579
JJA_spj_1948_(14)              QNYLPNTSENTYNSLQVSLVGVLLLFISMVNILWAKKSK     579
R6_spr1771_(2)                 QNYLPNTSENTYNSLQVSLVGVLLLFISMVNILWAKKSK     579
R6_E5Q10_09305_CIB17_(2)       QNYLPNTSENTYNSLQVSLVGVLLLFISMVNILWAKKSK     579
```

Figure S2. Multiple sequence alignment comparisons of SFP were performed using the bioinformatics tool "Clustal Omega" available on the following webpage: https://www.ebi.ac.uk/Tools/msa/clustalo/. The signal peptide is shown in red color.

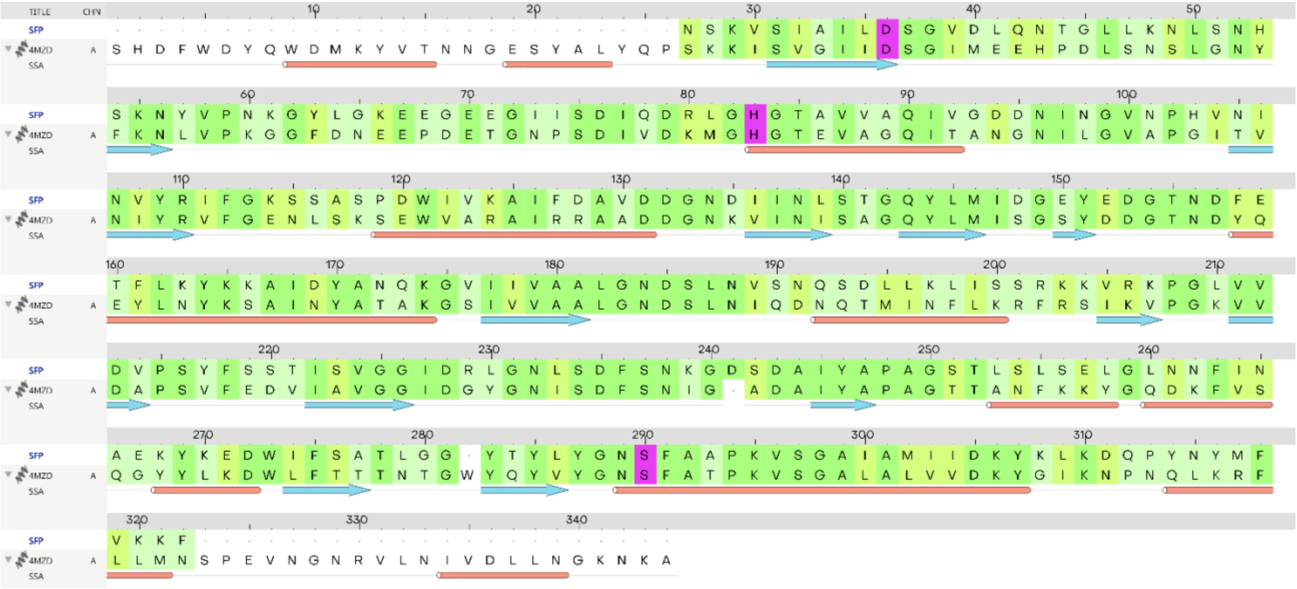

Figure S3. Sequence alignment of SFP catalytic domain (296 aa) with the corresponding template protein of the nisin leader peptidase NisP from *Lactococcus lactis* (4MZD) used for homology modeling (identical: green, similar: yellow, dissimilar: light green). The catalytic residues histidine (H), aspartate (D) and serine (S) are highlighted in purple. Red tubes and blue arrows depict helix and sheet secondary structures, respectively.

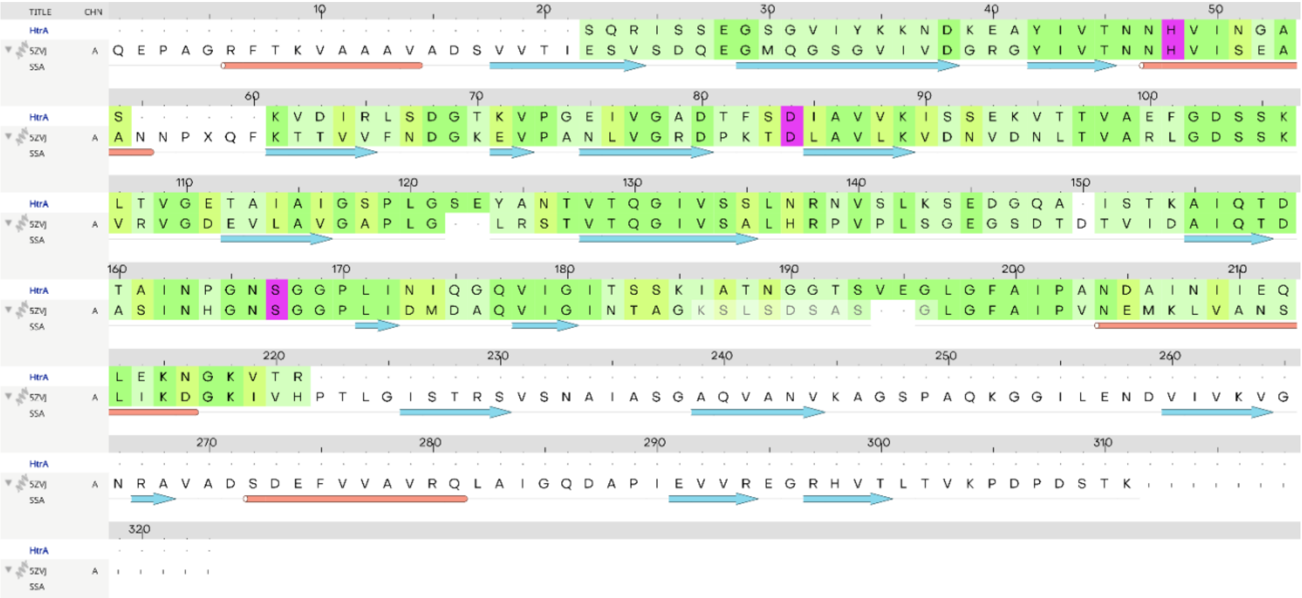

Figure S4. Sequence alignment of HtrA catalytic domain (182 aa) with the corresponding template protein of HtrA1 from *Mycobacterium tuberculosis* (5ZVJ) used for homology modeling (identical: green, similar: yellow, dissimilar: light green). The catalytic residues histidine (H), aspartate (D) and serine (S) are highlighted in purple. Red tubes and blue arrows depict helix and sheet secondary structures, respectively.

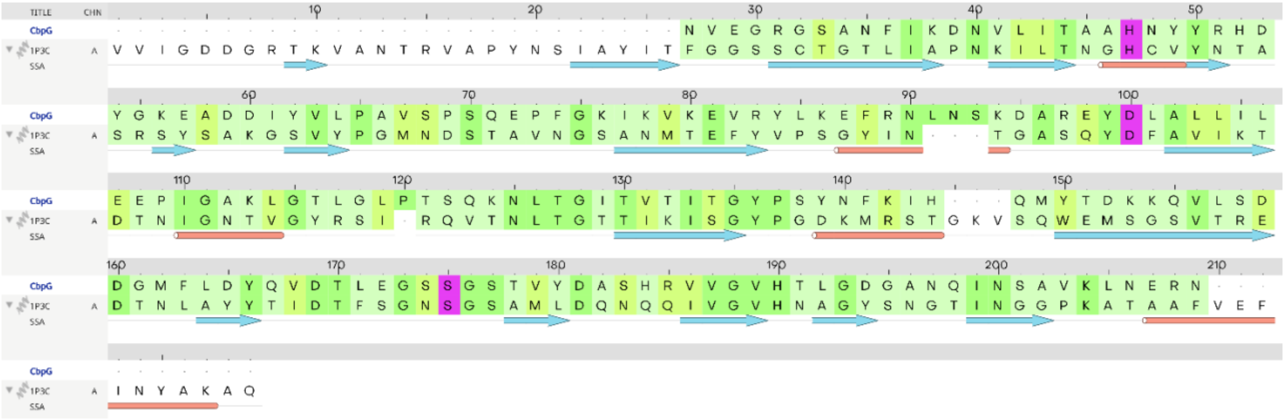

Figure S5. Sequence alignment of CbpG catalytic domain (184 aa) with the corresponding template protein glutamyl endopeptidase (1P3C) from *Bacillus intermedius* used for homology modeling (identical: green, similar: yellow, dissimilar: light green). The catalytic residues histidine (H), aspartate (D) and serine (S) are highlighted in purple. Red tubes and blue arrows depict helix and sheet secondary structures, respectively.

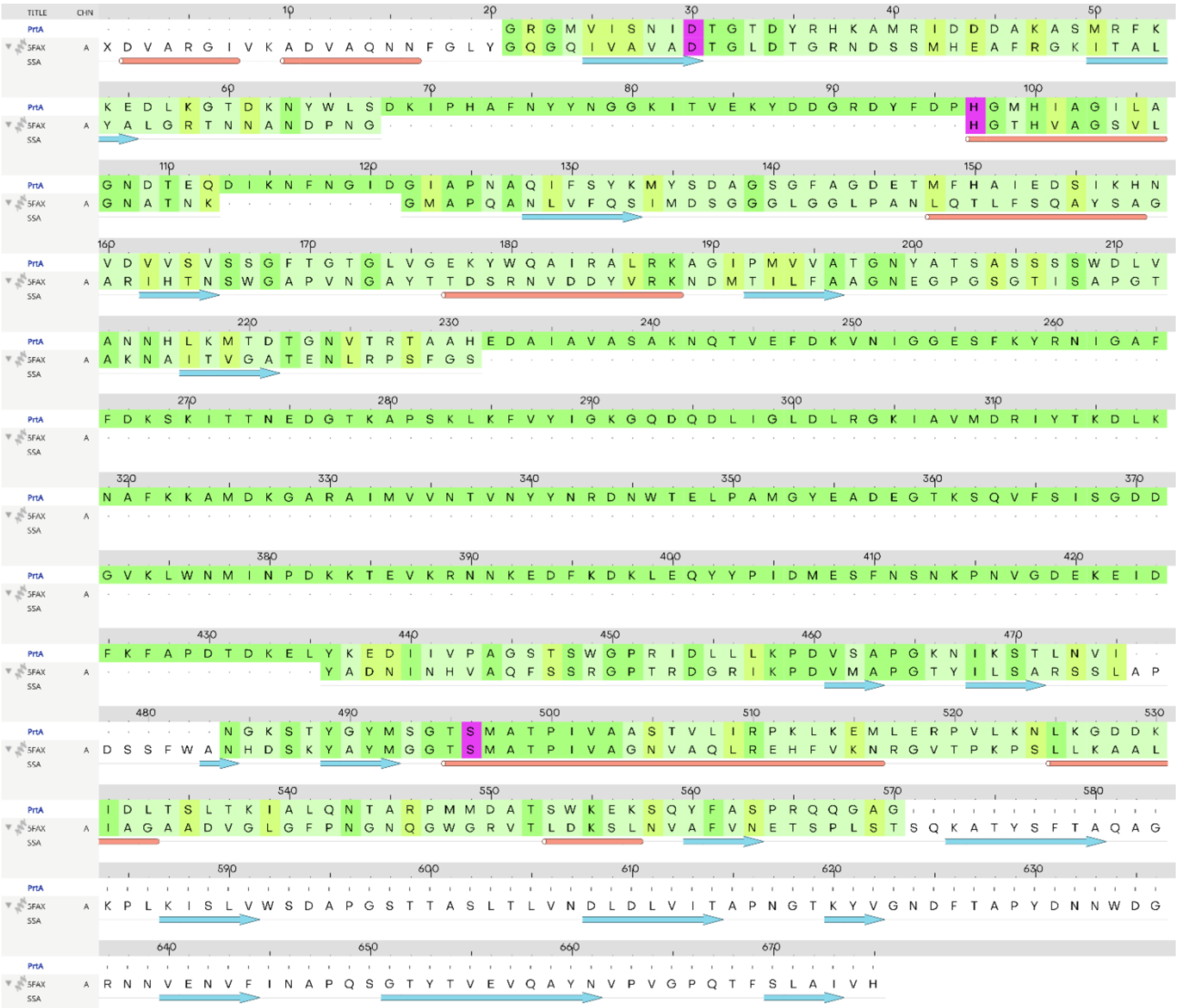

Figure S6. Sequence alignment of PrtA catalytic domain (542aa) with the corresponding template protein structure of subtilase SubHal from *Bacillus halmapalus* (5FAX) used for homology modeling (identical: green, similar: yellow, dissimilar: light green). The catalytic residues histidine (H), aspartate (D) and serine (S) are highlighted in purple. Red tubes and blue arrows depict helix and sheet secondary structures, respectively.
